# Supplementary material for: Tissue-adjusted pathway analysis of cancer (TPAC): A novel approach for quantifying tumor-specific gene set dysregulation relative to normal tissue
Source: PLoS Comput Biol. 2024 Jan 11;20(1):e1011717. doi: 10.1371/journal.pcbi.1011717 (PMC10807770; doi:10.1371/journal.pcbi.1011717)
Supplement: S1 Text — (PDF) [file pcbi.1011717.s001.pdf]

Supporting Information: Tissue-adjusted pathway analysis of cancer (TPAC): a novel approach for quantifying tumor-specific pathway dysregulation relative to normal tissue

H. Robert Frost

Contents

1 Supplemental Methods **2**

1.1 Cancer and normal tissue transcriptomic data 2

1.2 Alternative cancer outcomes 3

1.3 Gene sets 3

2 Supplemental Results **5**

List of Tables

A **The 21 analyzed TCGA cancer types, number of tumor samples with expression data, mean age, gender proportions, and corresponding HPA normal tissues. Note that gender proportions for some cancers may not add up to 1.0 if gender is unavailable for some samples.** 4

B Transcription factors whose activity scores for tumors in the TCGA KIRP cohort have the largest positive and negative rank correlation with overall TPAC scores for the MSigDB Hallmark MYC Targets V1 pathway. 16

List of Figures

A Heatmap illustrating the pan-cancer distribution of single sample GSVA scores for the MSigDB Hallmark pathways. 5

B Heatmap illustrating the pan-cancer distribution of single sample GRAPE scores for the MSigDB Hallmark pathways. 6

C Versions of the Q-Q plot in main manuscript Figure 3 that visualize the Cox model p-values for each TCGA cohort. 7

D Visualization of p-values and association direction for univariable Cox proportional hazards models fit using TPAC-generated scores for Hallmark pathways as single predictors and PFI as the outcome. Each cell is colored according to the magnitude of the -log(p-value) from the Cox model with positive values for hazard ratios >= 1 and negative values for hazard ratios < 1. 8

E Versions of the Q-Q plot in main manuscript Figure 4 that visualize tumor stage association p-values for each TCGA cohort. 9

F Visualization of p-values and association direction for Wilcoxon rank sum tests comparing TPAC scores for tumors with stage T01 vs. the scores for tumors with higher stages. Each cell is colored according to the magnitude of the -log(p-value) from the with positive values for cases where larger TPAC scores are associated with more advanced tumor stages and negative values where larger TPAC scores are associated with less severe tumor stages. . . . 10
G Versions of the Q-Q plot in main manuscript Figure 5 that visualize lymph node stage association p-values for each TCGA cohort. 11
H Visualization of p-values and association direction for Wilcoxon rank sum tests comparing TPAC scores for tumors associated with lymph node stage N0 vs. the scores for tumors associated with higher lymph node stages. Each cell is colored according to the magnitude of the -log(p-value) from the with positive values for cases where larger TPAC scores are associated with more advanced lymph node stages and negative values where larger TPAC scores are associated with less severe lymph node stages. 12
I Association between transcription factor (TF) activity, as estimated using the decoupleR method, and TPAC scores. Each cell represents the rank correlation between overall TPAC scores for one of the MSigDB Hallmark pathways and TF activity estimates across all analyzed TCGA cohorts. Results are only shown for the 25 TFs with the largest average absolute correlation. 13
J Association between transcription factor (TF) activity, as estimated using the decoupleR method, and TPAC scores. Each cell represents the rank correlation between overall TPAC scores for one of the MSigDB Hallmark pathways and TF activity estimates for the TCGA KIRP cohort. Results are only shown for the 25 TFs with the largest average absolute correlation. 14
K Association between transcription factor (TF) activity, as estimated using the decoupleR method, and TPAC scores. Each cell represents the rank correlation between overall TPAC scores for one of the MSigDB Hallmark pathways and TF activity estimates for the TCGA KIRC cohort. Results are only shown for the 25 TFs with the largest average absolute correlation. 15

1 Supplemental Methods

The TPAC R package and imported TPACData package are available on CRAN at https://cran.r-project.org/web/packa[illegible] and https://cran.r-project.org/web/packages/TPACData. A companion website for this paper can be found at https://hrfrost.host.dartmouth.edu/TPAC/. This site provides access to a version of the Human Protein Atlas (HPA) normal tissue gene expression data ("HPA.normal.FPKM.GDCpipeline.csv") that was specially normalized by the HPA group as FPKM using a pipeline similar to that employed by GDC for the TCGA data (this data was generated for the "Human Pathology Atlas" paper [1]). A summarized version of the HPA data is integrated into the TPACData package for use with the TPAC function *tpacForCancer()*. Please see the TPAC package documentation for a complete list of the supported functions, usage examples for those functions and a vignette illustrating the application of TPAC to TCGA liver cancer RNA-seq data using the MSigDB Hallmark gene sets.

1.1 Cancer and normal tissue transcriptomic data

Analysis results for 21 solid human cancers and the 18 associated normal tissues were generated using RNA-sequencing data from The Cancer Genome Atlas (TCGA) [2] and RNA-sequencing data from the Human Protein Atlas (HPA) [3]. Table A below lists the 21 supported TCGA cancer types, number of tumor samples, matching HPA cancer types and associated HPA normal human tissue types. As detailed in the HPA paper [3], the HPA RNA-seq measurements were made on frozen tissue sections from the Uppsala Biobank for three healthy individuals. These 21 cancer types were selected based on the availability of

gene expression data for the corresponding normal tissues in the HPA. A similar set of 17 TCGA cancer types were selected for the analysis by Uhlen et al. [1]. In contrast to Uhlen et al., we have separately analyzed the three renal cancer types (kidney chromophobe, kidney renal clear cell carcinoma, and kidney renal papillary cell carcinoma), separately analyzed colon cancer and rectal cancer, and separately analyzed lung adenocarcinoma and lung squamous cell carcinoma. RNA-seq data was filtered to include just the 19,670 genes with measurements on all normal tissues and cancer types and was normalized as FPKM $+1$ using the Genome Data Commons (GDC) pipeline for the TCGA data and using a pipeline similar to GDC pipeline for the HPA data.

- For TCGA, the PANCAN RNA-seq and phenotype data contained in the files "GDC-PANCAN.htseq_fpkm.tsv.gz" and "GDC-PANCAN.GDC_phenotype.tsv" was downloaded from the GCD Data Portal (https://gdc.cancer.gov/). Note that an equivalent FPKM-normalized version of the TCGA RNA-seq data can be access from the HPA project at https://www.proteinatlas.org/download/rna_cancer_sample.tsv.zip.
- Alternative cancer outcomes (e.g., progression-free interval) were accessed from the TCGA Pan-Cancer Clinical Data Resource (https://gdc.cancer.gov/about-data/publications/PanCan-Clinical-2018).
- For HPA, the HPA staff provided normal tissue gene expression data in the file "HPA.normal.FPKM.GDCpipeline.csv"; this data was specially normalized by the HPA group as FPKM using a pipeline similar to that employed by GDC for the TCGA data (this data was generated for the "Human Pathology Atlas" paper [1]).
  A copy of this file can be retrieved from the paper website (https://hrfrost.host.dartmouth.edu/TPAC/).

Note: while the results in this paper were generated using FPKM-normalized version of the TCGA and HPA RNA-seq data (in part for consistency with the prior papers by the HPA group [1] and our lab [4]), the TPAC method can be applied to other cancer and normal tissue transcriptomic datasets as long as equivalent processing pipelines are used (i.e., aligned to the same reference genome, same normalization method, etc.).

1.2 Alternative cancer outcomes

Alternative cancer outcomes (e.g., progression-free interval) were accessed from the TCGA Pan-Cancer Clinical Data Resource (TCGA-CDR) [5] at https://gdc.cancer.gov/about-data/publications/PanCan-Clinical-2018,

1.3 Gene sets

The Hallmark collection gene sets were downloaded from version 7.2 of the Molecular Signatures Database (MSigDB) [6] (as downloaded from http://software.broadinstitute.org/gsea/downloads.jsp).

TCGA abbrev.	# TCGA samples	Mean TCGA age at diagnosis (sd)	TCGA male/female proportions	HPA cancer type	HPA tissue
BLCA	430	68.2 (10.5)	0.73/0.27	Bladder Urothelial Carcinoma	urinary bladder
BRCA	1,217	58.3 (13.4)	0.01/0.99	Breast Invasive Carcinoma	breast
CESC	309	48.2 (13.7)	0/1	Cervical Squamous Cell Carcinoma and ...	cervix, uterine
COAD	512	67.6 (13.0)	0.52/0.48	Colon Adenocarcinoma	colon
GBM	173	57.7 (14.3)	0.60/0.37	Glioblastoma Multiforme	cerebral cortex
HNSC	546	61.0 (11.9)	0.73/0.27	Head and Neck Squamous Cell Carcinoma	
KICH	89	51.9 (14.3)	0.55/0.45	Kidney Chromophobe	kidney
KIRC	607	60.8 (12.1)	0.65/0.35	Kidney Renal Clear Cell Carcinoma	kidney
KIRP	321	62.0 (12.2)	0.74/0.26	Kidney Renal Papillary Cell Carcinoma	kidney
LIHC	424	60.3 (13.8)	0.66/0.34	Liver Hepatocellular Carcinoma	liver
LUAD	585	65.2 (10.1)	0.39/0.47	Lung Adenocarcinoma	lung
LUSC	550	67.3 (8.7)	0.73/0.27	Lung Squamous Cell Carcinoma	lung
OV	379	59.6 (11.4)	0/0.97	Ovarian Serous Cystadenocarcinoma	ovary
PAAD	182	64.9 (11.4)	0.56/0.44	Pancreatic Adenocarcinoma	pancreas
PRAD	551	60.9 (6.8)	1/0	Prostate Adenocarcinoma	prostate
READ	177	64.2 (12.2)	0.52/0.46	Rectum Adenocarcinoma	rectum
SKCM	472	58.2 (15.6)	0.62/0.38	Skin Cutaneous Melanoma	skin
STAD	407	66.0 (10.7)	0.63/0.37	Stomach Adenocarcinoma	stomach
TGCT	156	31.9 (9.2)	0.89/0	Testicular Germ Cell Tumors	testis
THCA	568	47.2 (15.8)	0.27/0.73	Thyroid Carcinoma	thyroid gland
UCEC	583	70.1 (9.6)	0/1	Uterine Corpus Endometrial Carcinoma	endometrium

Table A: **The 21 analyzed TCGA cancer types, number of tumor samples with expression data, mean age, gender proportions, and corresponding HPA normal tissues. Note that gender proportions for some cancers may not add up to 1.0 if gender is unavailable for some samples.**

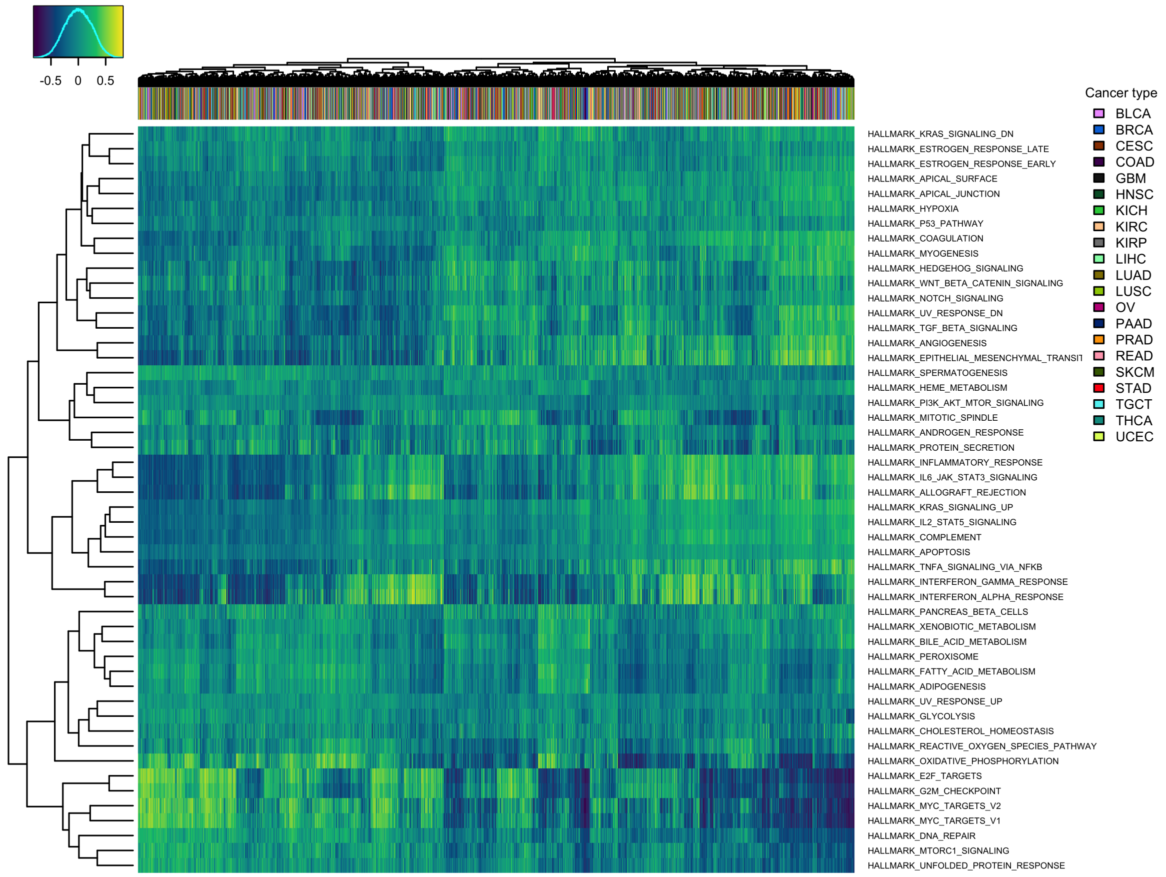

Figure A: Heatmap illustrating the pan-cancer distribution of single sample GSVA scores for the MSigDB Hallmark pathways.

2 Supplemental Results

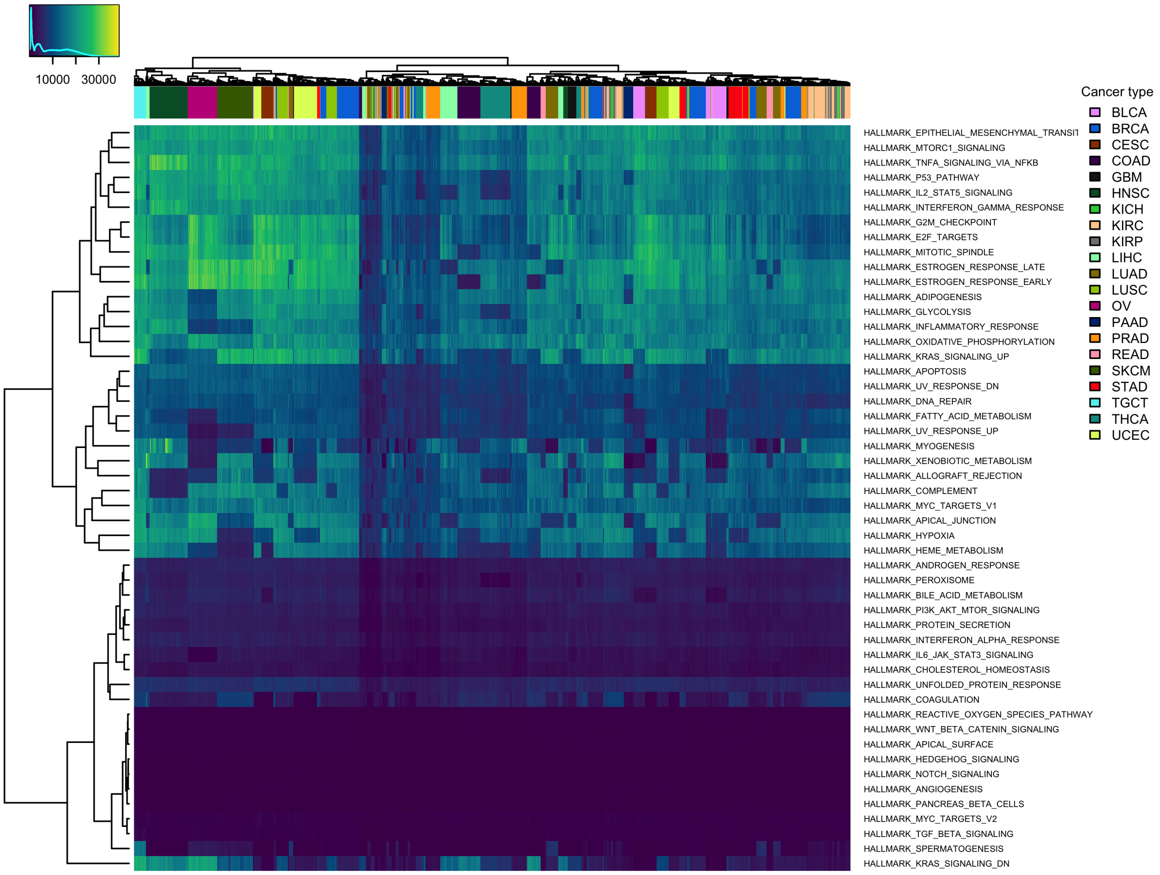

Figure B: Heatmap illustrating the pan-cancer distribution of single sample GRAPE scores for the MSigDB Hallmark pathways.

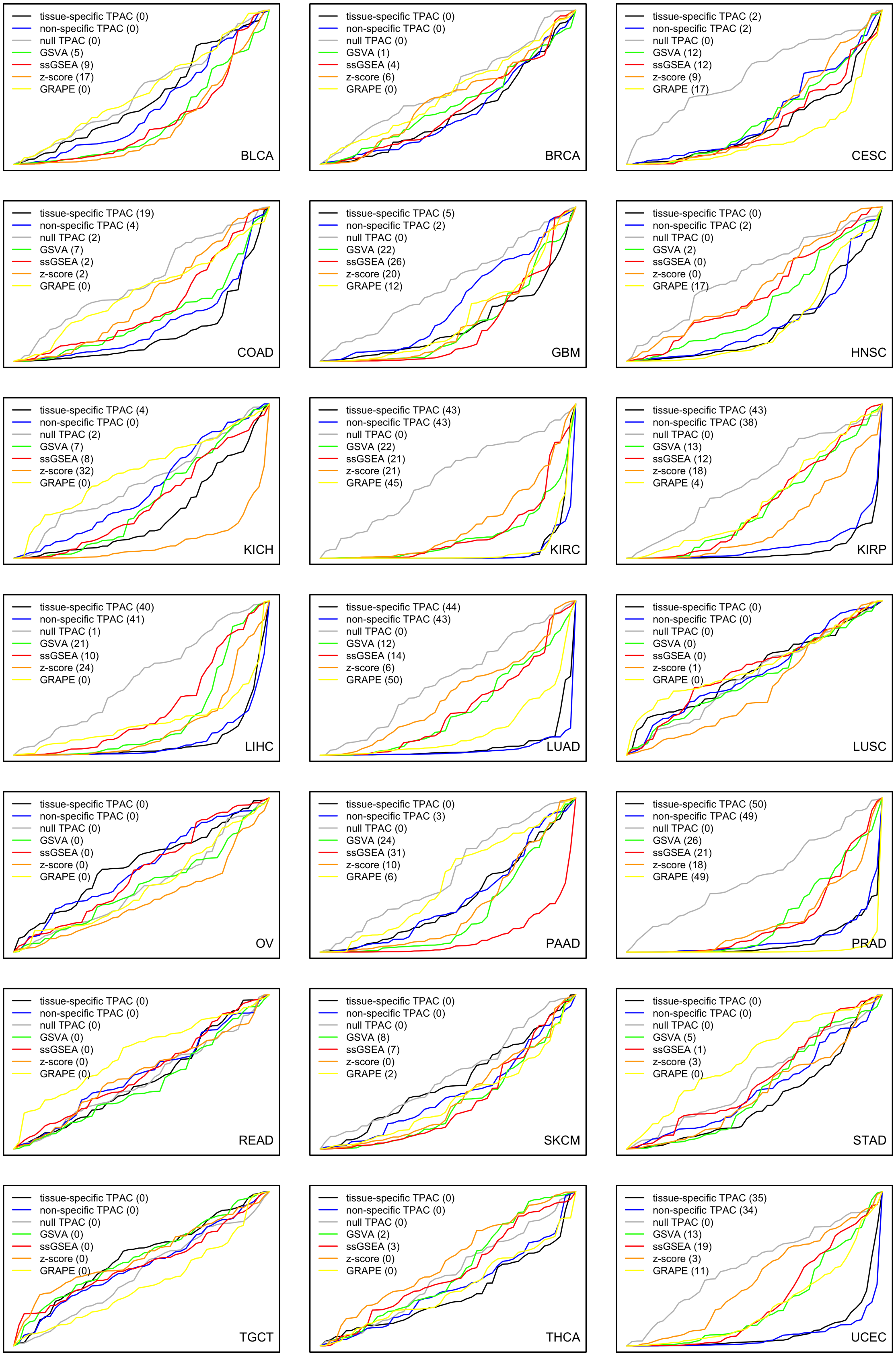

Figure C: Versions of the Q-Q plot in main manuscript Figure 3 that visualize the Cox model p-values for each TCGA cohort.

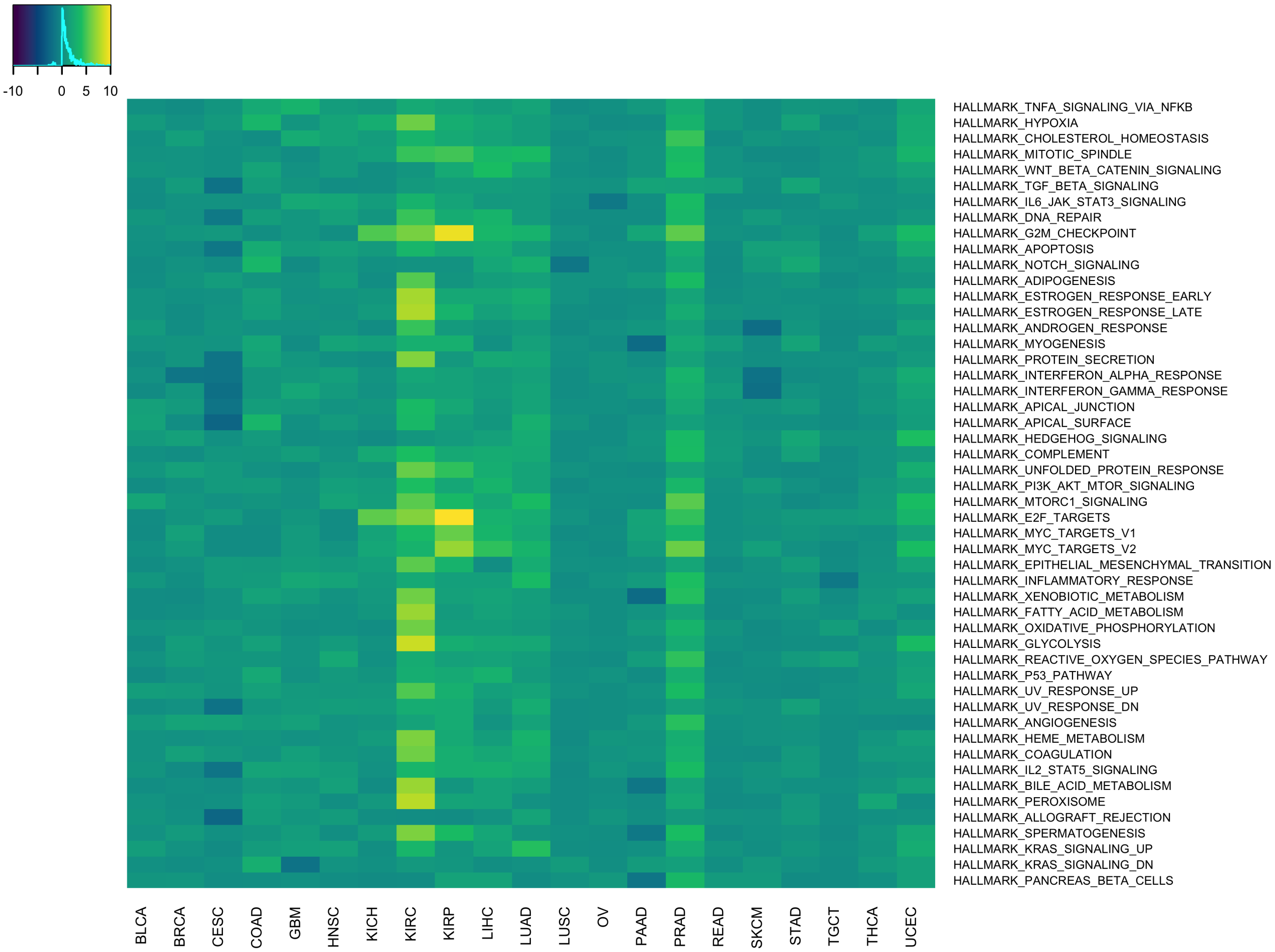

Figure D: Visualization of p-values and association direction for univariable Cox proportional hazards models fit using TPAC-generated scores for Hallmark pathways as single predictors and PFI as the outcome. Each cell is colored according to the magnitude of the -log(p-value) from the Cox model with positive values for hazard ratios >= 1 and negative values for hazard ratios < 1.

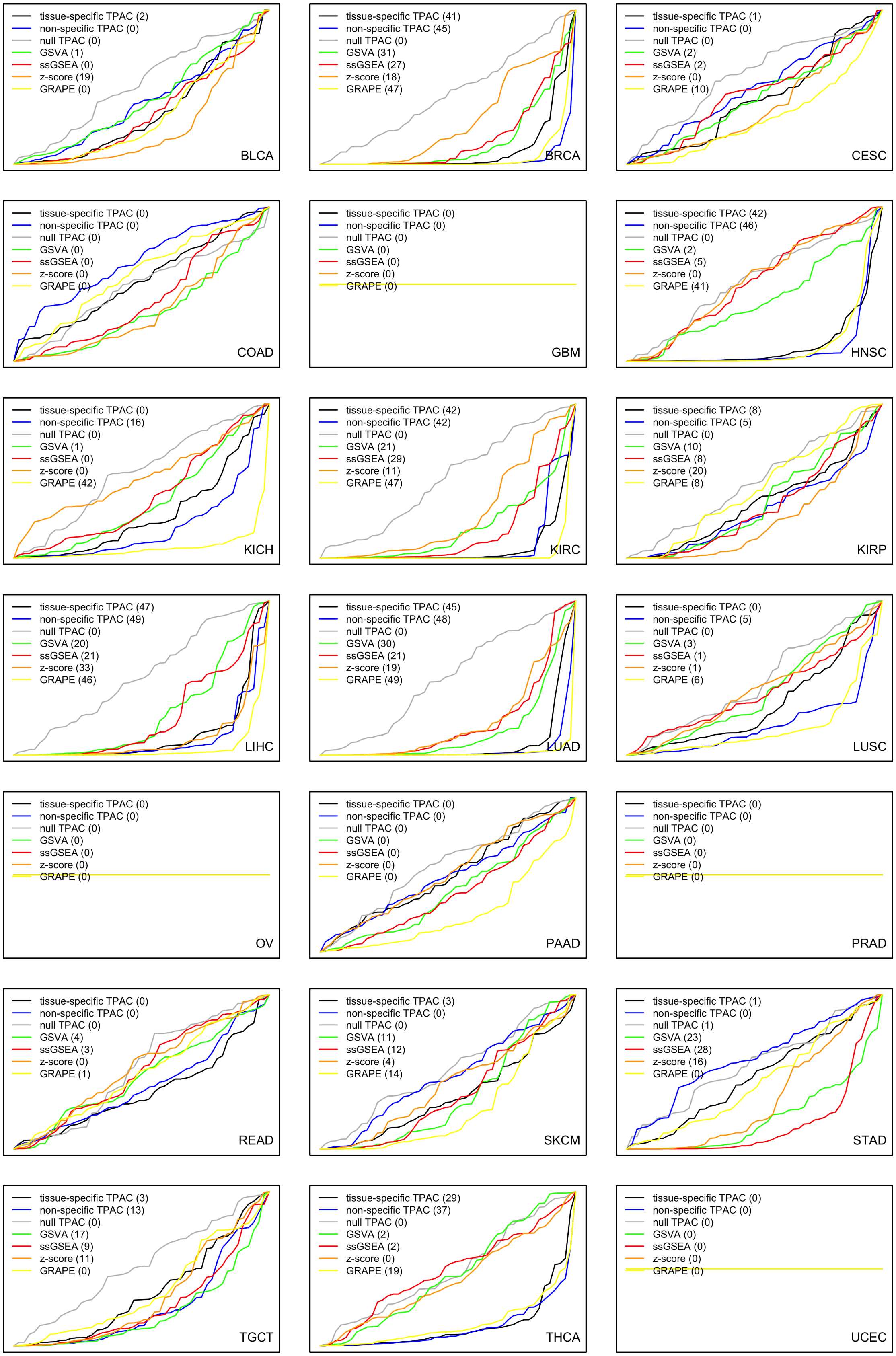

Figure E: Versions of the Q-Q plot in main manuscript Figure 4 that visualize tumor stage association p-values for each TCGA cohort.

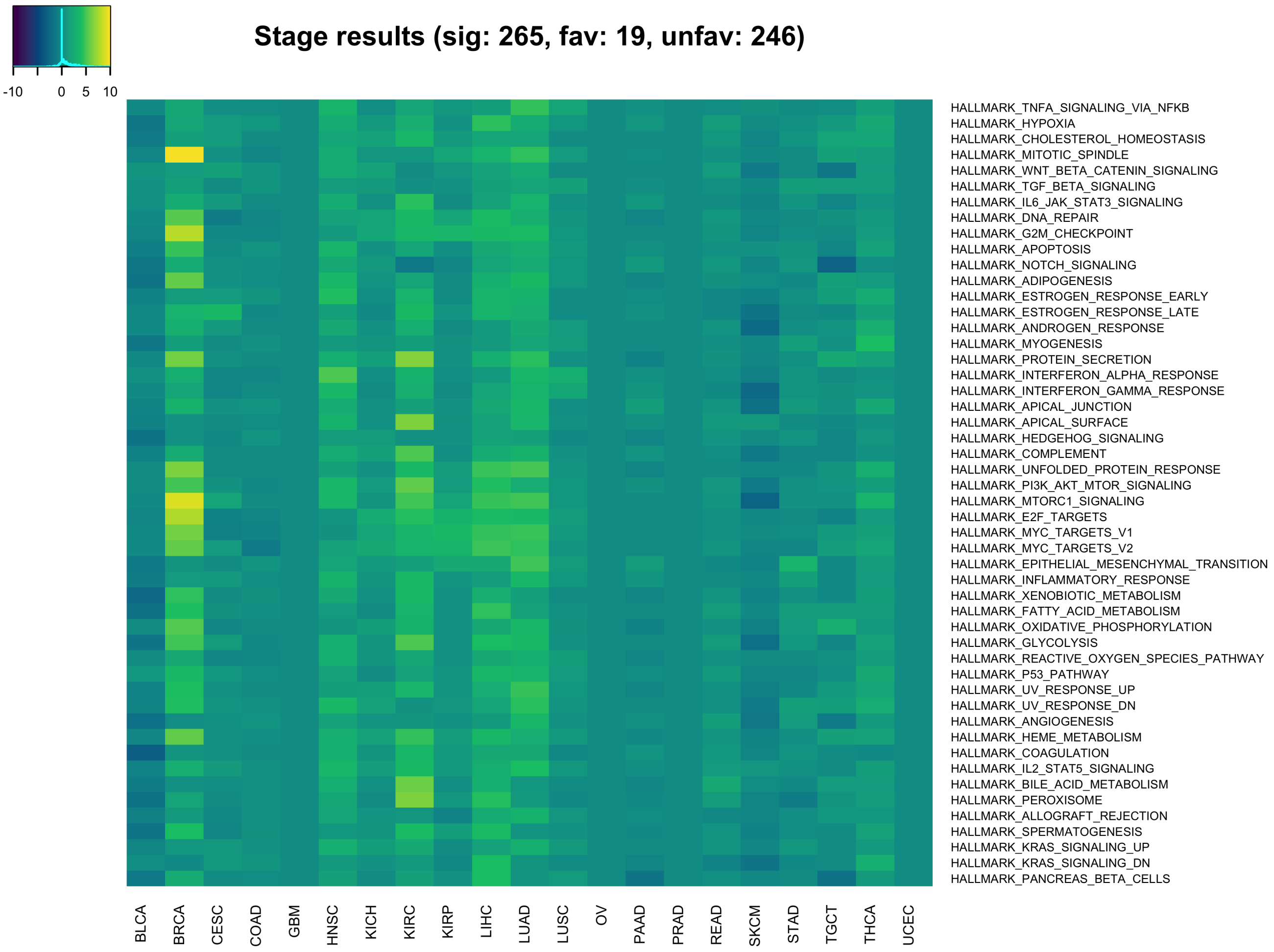

Figure F: Visualization of p-values and association direction for Wilcoxon rank sum tests comparing TPAC scores for tumors with stage T01 vs. the scores for tumors with higher stages. Each cell is colored according to the magnitude of the -log(p-value) from the with positive values for cases where larger TPAC scores are associated with more advanced tumor stages and negative values where larger TPAC scores are associated with less severe tumor stages.

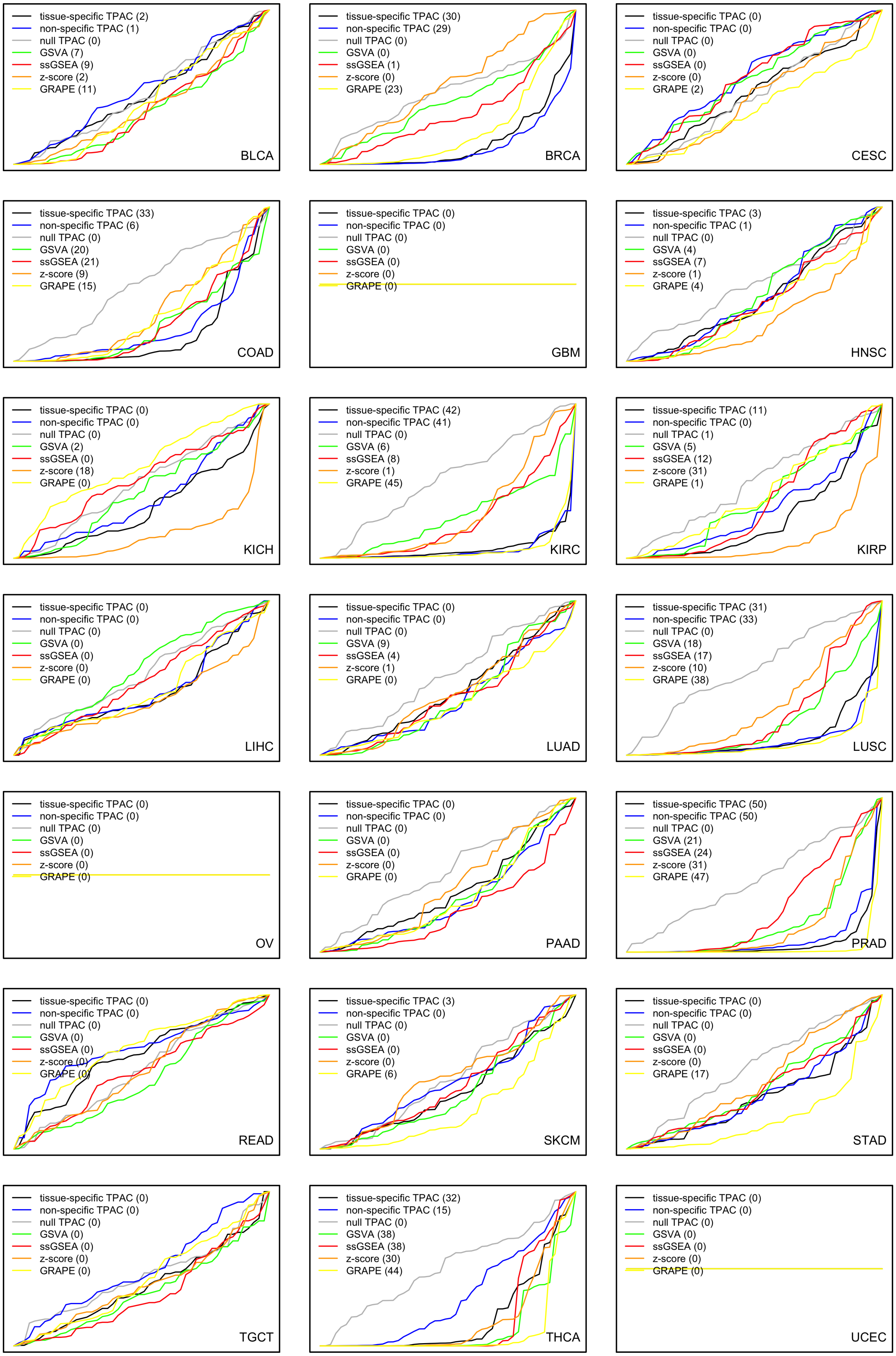

Figure G: Versions of the Q-Q plot in main manuscript Figure 5 that visualize lymph node stage association p-values for each TCGA cohort.

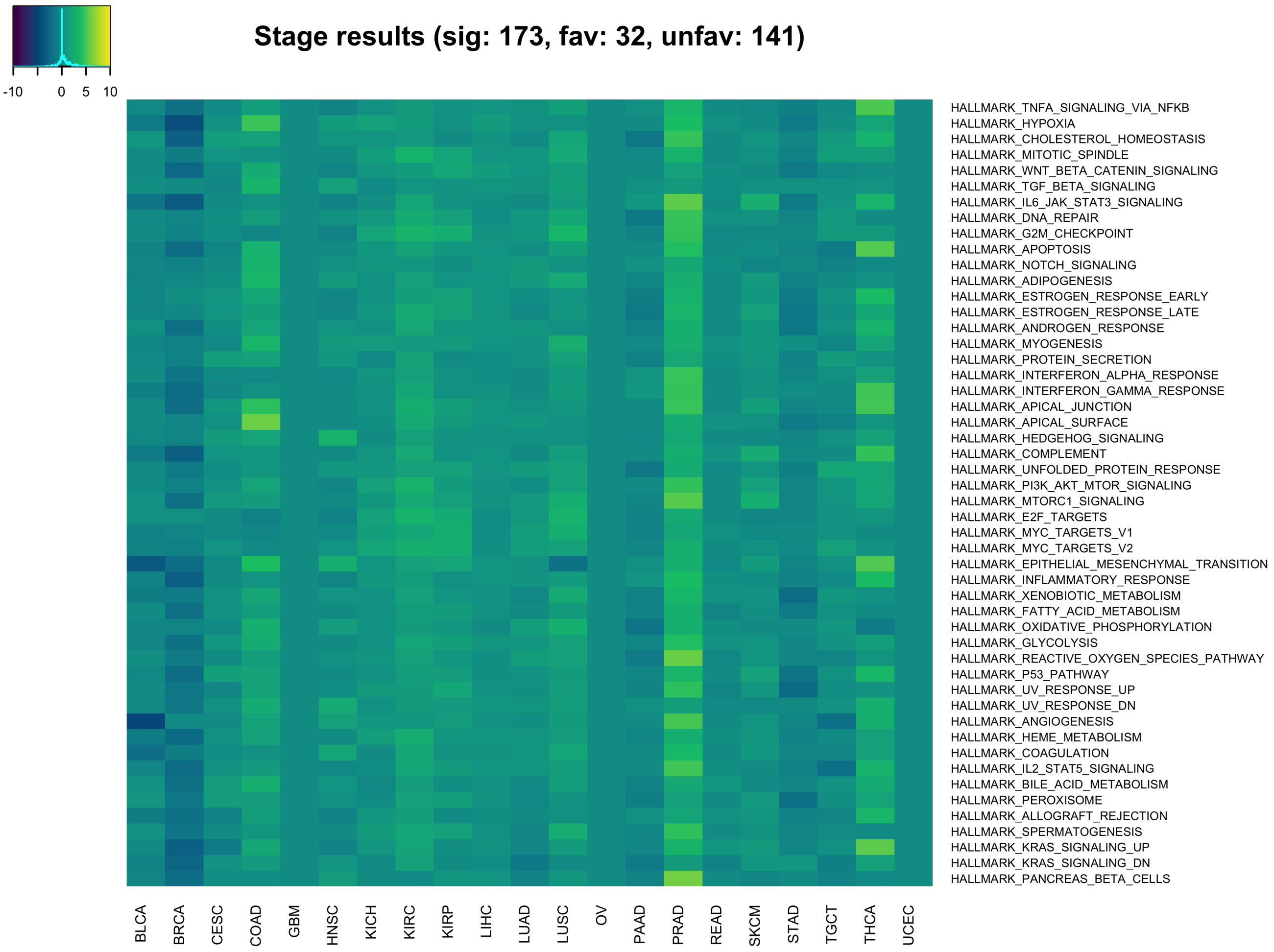

Figure H: Visualization of p-values and association direction for Wilcoxon rank sum tests comparing TPAC scores for tumors associated with lymph node stage N0 vs. the scores for tumors associated with higher lymph node stages. Each cell is colored according to the magnitude of the -log(p-value) from the with positive values for cases where larger TPAC scores are associated with more advanced lymph node stages and negative values where larger TPAC scores are associated with less severe lymph node stages.

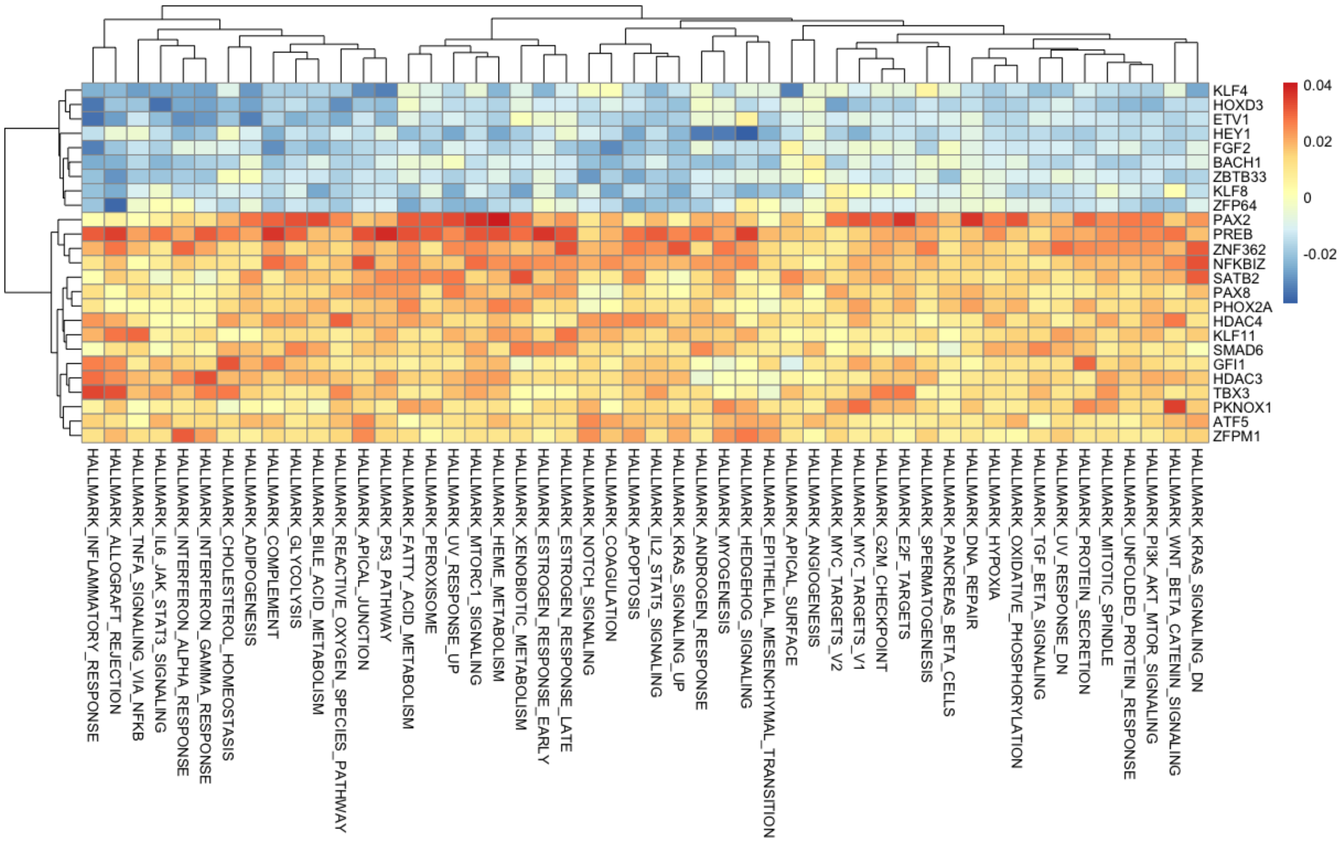

Figure I: Association between transcription factor (TF) activity, as estimated using the decoupleR method, and TPAC scores. Each cell represents the rank correlation between overall TPAC scores for one of the MSigDB Hallmark pathways and TF activity estimates across all analyzed TCGA cohorts. Results are only shown for the 25 TFs with the largest average absolute correlation.

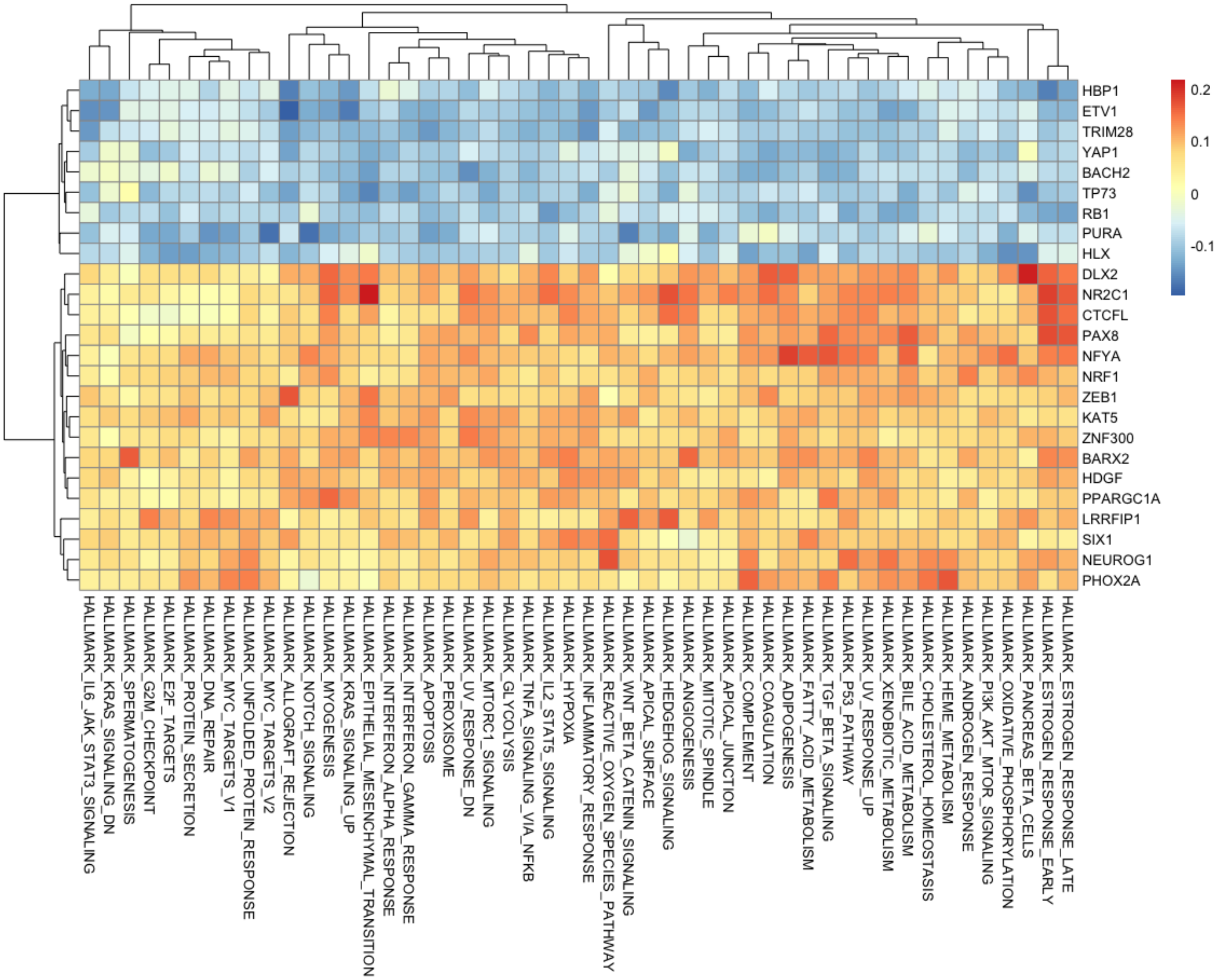

Figure J: Association between transcription factor (TF) activity, as estimated using the decoupleR method, and TPAC scores. Each cell represents the rank correlation between overall TPAC scores for one of the MSigDB Hallmark pathways and TF activity estimates for the TCGA KIRP cohort. Results are only shown for the 25 TFs with the largest average absolute correlation.

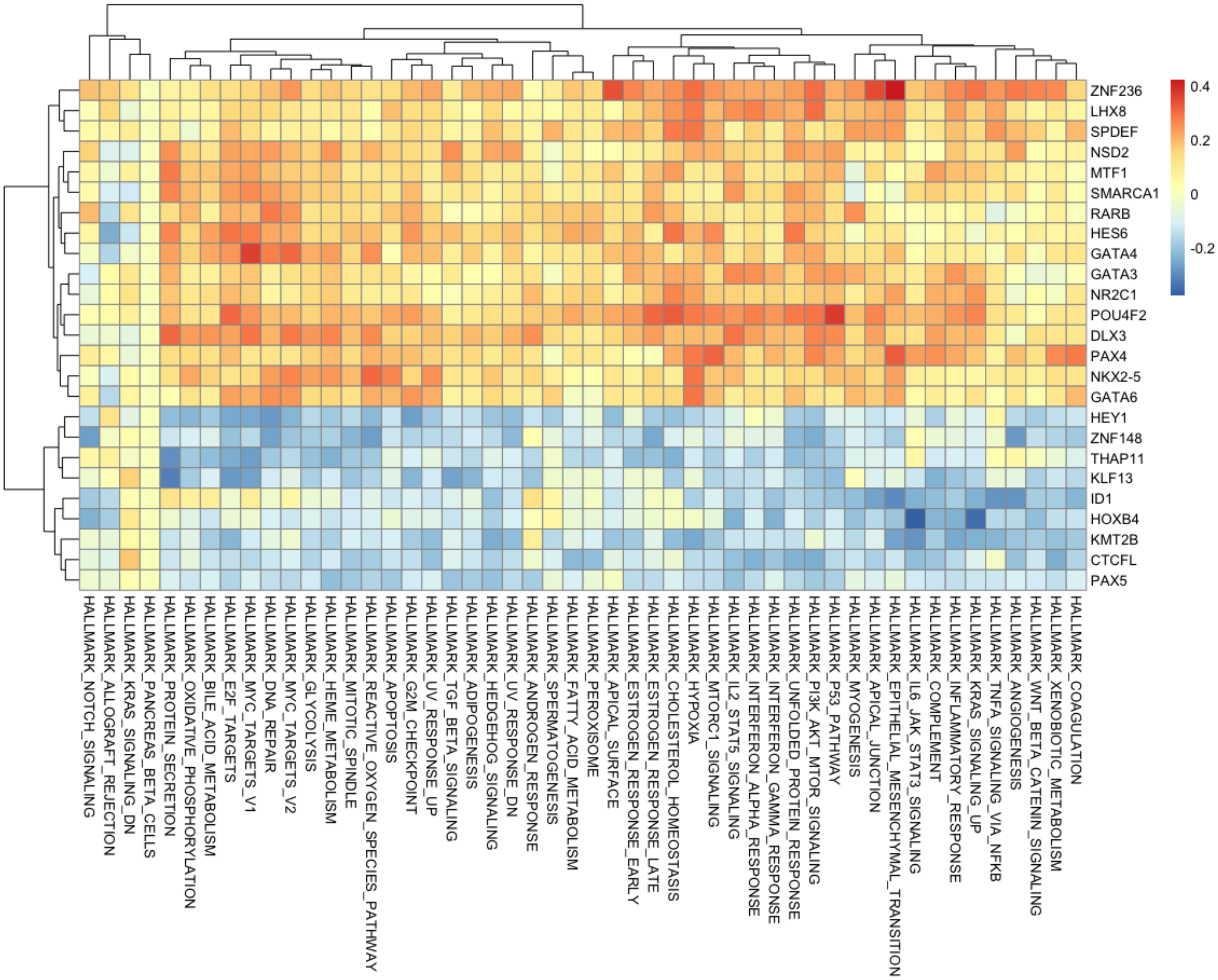

Figure K: Association between transcription factor (TF) activity, as estimated using the decoupleR method, and TPAC scores. Each cell represents the rank correlation between overall TPAC scores for one of the MSigDB Hallmark pathways and TF activity estimates for the TCGA KIRC cohort. Results are only shown for the 25 TFs with the largest average absolute correlation.

TF	ρ	TF	ρ
SIX4	0.138	PURA	-0.135
PHOX2A	0.136	HLX	-0.116
LRRFIP1	0.126	MYRF	-0.114
NEUROG1	0.114	HOXA7	-0.113
NRF1	0.098	NR3C2	-0.106

Table B: Transcription factors whose activity scores for tumors in the TCGA KIRP cohort have the largest positive and negative rank correlation with overall TPAC scores for the MSigDB Hallmark MYC Targets V1 pathway.

References

[1] Uhlen, M., Zhang, C., Lee, S., Sjöstedt, E., Fagerberg, L., Bidkhori, G., Benfeitas, R., Arif, M., Liu, Z., Edfors, F., Sanli, K., von Feilitzen, K., Oksvold, P., Lundberg, E., Hober, S., Nilsson, P., Mattsson, J., Schwenk, J.M., Brunnström, H., Glimelius, B., Sjöblom, T., Edqvist, P.-H., Djureinovic, D., Micke, P., Lindskog, C., Mardinoglu, A., Ponten, F.: A pathology atlas of the human cancer transcriptome. Science **357**(6352) (2017). doi:10.1126/science.aan2507

[2] Cancer Genome Atlas Research Network, Weinstein, J.N., Collisson, E.A., Mills, G.B., Shaw, K.R.M., Ozenberger, B.A., Ellrott, K., Shmulevich, I., Sander, C., Stuart, J.M.: The cancer genome atlas pan-cancer analysis project. Nat Genet **45**(10), 1113–20 (2013). doi:10.1038/ng.2764

[3] Uhlén, M., Fagerberg, L., Hallström, B.M., Lindskog, C., Oksvold, P., Mardinoglu, A., Sivertsson, Å., Kampf, C., Sjöstedt, E., Asplund, A., Olsson, I., Edlund, K., Lundberg, E., Navani, S., Szigyarto, C.A.-K., Odeberg, J., Djureinovic, D., Takanen, J.O., Hober, S., Alm, T., Edqvist, P.-H., Berling, H., Tegel, H., Mulder, J., Rockberg, J., Nilsson, P., Schwenk, J.M., Hamsten, M., von Feilitzen, K., Forsberg, M., Persson, L., Johansson, F., Zwahlen, M., von Heijne, G., Nielsen, J., Pontén, F.: Proteomics. tissue-based map of the human proteome. Science **347**(6220), 1260419 (2015). doi:10.1126/science.1260419

[4] Frost, H.R.: Analyzing cancer gene expression data through the lens of normal tissue-specificity. PLoS Comput Biol **17**(6), 1009085 (2021). doi:10.1371/journal.pcbi.1009085

[5] Liu, J., Lichtenberg, T., Hoadley, K.A., Poisson, L.M., Lazar, A.J., Cherniack, A.D., Kovatich, A.J., Benz, C.C., Levine, D.A., Lee, A.V., Omberg, L., Wolf, D.M., Shriver, C.D., Thorsson, V., Cancer Genome Atlas Research Network, Hu, H.: An integrated tcga pan-cancer clinical data resource to drive high-quality survival outcome analytics. Cell **173**(2), 400–41611 (2018). doi:10.1016/j.cell.2018.02.052

[6] Liberzon, A., Subramanian, A., Pinchback, R., Thorvaldsdóttir, H., Tamayo, P., Mesirov, J.P.: Molecular signatures database (msigdb) 3.0. Bioinformatics **27**(12), 1739–40 (2011). doi:10.1093/bioinformatics/btr260
