# Supplementary material for: Tissue-adjusted pathway analysis of cancer (TPAC): A novel approach for quantifying tumor-specific gene set dysregulation relative to normal tissue
Source: PLoS Comput Biol. 2024 Jan 11;20(1):e1011717. doi: 10.1371/journal.pcbi.1011717 (PMC10807770; doi:10.1371/journal.pcbi.1011717)
Supplement: S2 Text — (PDF) [file pcbi.1011717.s002.pdf]

Example analysis of TCGA liver cancer RNA-seq data using TPAC R package

H. Robert Frost

The following vignette leverages the TPAC R package available from https://hrfrost.host.dartmouth.edu/TPAC/ to analyze TCGA liver cancer RNA-seq data. This logic is based on the vignette included in the R package.

1 Load and process TCGA liver cancer RNA-seq data

The following logic loads FPKM normalized counts for The Cancer Genome Atlas (TCGA) [1] liver cancer (LIHC) cohort. The TPAC function *tpacForCancer()* leverages Human Protein Atlas (HPA) normal tissue gene expression data ("HPA.normal.FPKM.GDCpipeline.csv") that was specially processed by the HPA group as FPKM values using a pipeline similar to that employed by GDC for the TCGA data (this data was generated for the "Human Pathology Atlas" paper [2]). For consistency with this HPA normal tissue data, the TCGA data is retrieved from the HPA provided TCGA gene expression data file rna_cancer_sample.tsv, which contains FPKM normalized counts and can be downloaded from https://www.proteinatlas.org/download/rna_cancer_sample.tsv.zip.
Generation of the LIHC-specific matrix from the rna_cancer_sample.tsv data was performed using the following R code (which is not executed here given the size of the data and processing time):

```
library(data.table)
library(reshape2)
tcga.file = "rna_cancer_sample.tsv"
data = read.table(file=tcga.file, header=T, sep="\t", stringsAsFactors=F,
                  # Columns: "Gene", "Sample", "Cancer", "FPKM"
                  colClasses=c("factor", "factor", "factor", "numeric"))
data = as.data.table(data)
setkey(data, Cancer, Gene)
lihc.data = data[.("LIHC")]
lihc.matrix = acast(lihc.data, Sample~Gene, value.var="FPKM")
saveRDS(lihc.matrix, file="lihc.rna.matrix.rds")
```

```
> # read in saved matrix
> liver.counts.fpkm = readRDS(file="lihc.rna.matrix.rds")
> # change to FPKM + 1
> liver.counts.fpkm = liver.counts.fpkm + 1
```

2 Load the MSigDB Hallmark collection

The following logic loads the MSigDB Hallmark collection using the msigdbr R package. The data frame returned by msigdbr is then converted into a list of gene ID vectors (each list element corresponds to a gene set and is a vector of Ensembl IDs). The *tpacForCancer()* function automatically transforms this into a list of vectors of gene indices using the *createGeneSetCollection()* helper function.

```
> # Load the MSigDB Hallmark collection using the msigdbr package
> hallmark.collection = msigdbr::msigdbr(category="H")
> # Create a gene.set.collection list of Ensembl IDs
> gene.set.names = unique(hallmark.collection$gs_name)
> num.sets = length(gene.set.names)
> message("Number of sets in MSigDB Hallmark collection: ", num.sets)
> gene.set.names[1:5]

[1] "HALLMARK_ADIPOGENESIS"        "HALLMARK_ALLOGRAFT_REJECTION"
[3] "HALLMARK_ANDROGEN_RESPONSE"   "HALLMARK_ANGIOGENESIS"
[5] "HALLMARK_APICAL_JUNCTION"

> gene.set.collection = list()
> for (i in 1:num.sets) {
+         gene.set.name = gene.set.names[i]
+         gene.set.rows = which(hallmark.collection$gs_name == gene.set.name)
+         gene.set.ensembl.ids = hallmark.collection$human_ensembl_gene[gene.set.rows]
+         gene.set.collection[[i]] = unique(gene.set.ensembl.ids)
+ }
> names(gene.set.collection) = gene.set.names
```

3 Execute TPAC method

Since we are processing TCGA RNA-seq liver cancer data, we can execute TPAC using the *tpacForCancer()* wrapper function. Note that the cancer types supported by *tpacForCancer()* can be accessed via the *getSupportedCancerTypes()* function.

```
> # Display the full list of cancer types supported by tpacForCancer()
> TPAC::getSupportedCancerTypes()

 [1] "urothelial cancer"    "breast cancer"        "cervical cancer"
 [4] "colorectal cancer"    "glioma"               "head and neck cancer"
 [7] "renal cancer"         "liver cancer"         "lung cancer"
[10] "ovarian cancer"       "pancreatic cancer"    "prostate cancer"
[13] "colorectal cancer"    "melanoma"             "stomach cancer"
[16] "testis cancer"        "thyroid cancer"       "endometrial cancer"

> # Get the normal tissue corresponding to liver cancer
> cancer.type = "liver cancer"
> # Execute TPAC
> tpac.out = TPAC::tpacForCancer(cancer.gene.expr=liver.counts.fpkm,
+                              cancer.type=cancer.type,
+                              gene.set.collection=gene.set.collection)
```

Look at a subset of the TPAC scores in the generated S, S- and S+ matrices:

```
> tpac.out$S[1:5,1:5]

                 HALLMARK_ADIPOGENESIS HALLMARK_ALLOGRAFT_REJECTION
TCGA-2Y-A9GS-01A          2.719203e-03                 2.947274e-01
TCGA-2Y-A9GT-01A          5.762057e-14                 7.274027e-05
TCGA-2Y-A9GU-01A          9.910067e-01                 8.682998e-01
```

```
TCGA-2Y-A9GV-01A          0.000000e+00                1.561800e-04
TCGA-2Y-A9GW-01A          5.681916e-01                3.033752e-03
                 HALLMARK_ANDROGEN_RESPONSE HALLMARK_ANGIOGENESIS
TCGA-2Y-A9GS-01A               2.480038e-01          0.7588796353
TCGA-2Y-A9GT-01A               3.029263e-06          0.9999999841
TCGA-2Y-A9GU-01A               4.754157e-01          0.0003233058
TCGA-2Y-A9GV-01A               6.604964e-04          0.9997427801
TCGA-2Y-A9GW-01A               9.106814e-01          0.9999999999
                 HALLMARK_APICAL_JUNCTION
TCGA-2Y-A9GS-01A               0.23158354
TCGA-2Y-A9GT-01A               0.02765183
TCGA-2Y-A9GU-01A               0.13971502
TCGA-2Y-A9GV-01A               0.04910314
TCGA-2Y-A9GW-01A               0.39696742
```

```
> tpac.out$S.neg[1:5,1:5]
```

```
                 HALLMARK_ADIPOGENESIS HALLMARK_ALLOGRAFT_REJECTION
TCGA-2Y-A9GS-01A          1.410908e-03                0.4469749673
TCGA-2Y-A9GT-01A          1.871836e-13                0.0002379945
TCGA-2Y-A9GU-01A          9.831630e-01                0.9612815610
TCGA-2Y-A9GV-01A          0.000000e+00                0.0010218391
TCGA-2Y-A9GW-01A          6.243827e-01                0.0087758806
                 HALLMARK_ANDROGEN_RESPONSE HALLMARK_ANGIOGENESIS
TCGA-2Y-A9GS-01A               2.698909e-01           0.850956347
TCGA-2Y-A9GT-01A               6.623778e-06           0.999999997
TCGA-2Y-A9GU-01A               5.387617e-01           0.001064207
TCGA-2Y-A9GV-01A               1.044634e-03           0.999859269
TCGA-2Y-A9GW-01A               9.276508e-01           1.000000000
                 HALLMARK_APICAL_JUNCTION
TCGA-2Y-A9GS-01A              0.041703909
TCGA-2Y-A9GT-01A              0.007417473
TCGA-2Y-A9GU-01A              0.566631061
TCGA-2Y-A9GV-01A              0.007196906
TCGA-2Y-A9GW-01A              0.140995454
```

```
> tpac.out$S.pos[1:5,1:5]
```

```
                 HALLMARK_ADIPOGENESIS HALLMARK_ALLOGRAFT_REJECTION
TCGA-2Y-A9GS-01A            0.85525943                 0.225709024
TCGA-2Y-A9GT-01A            0.08863464                 0.183958064
TCGA-2Y-A9GU-01A            0.96184567                 0.001469159
TCGA-2Y-A9GV-01A            0.36890422                 0.068879469
TCGA-2Y-A9GW-01A            0.16750628                 0.187664264
                 HALLMARK_ANDROGEN_RESPONSE HALLMARK_ANGIOGENESIS
TCGA-2Y-A9GS-01A                 0.37637394             0.1664063
TCGA-2Y-A9GT-01A                 0.03461565             0.6035765
TCGA-2Y-A9GU-01A                 0.02570467             0.3430984
TCGA-2Y-A9GV-01A                 0.11470915             0.8801873
TCGA-2Y-A9GW-01A                 0.21347707             0.3072954
                 HALLMARK_APICAL_JUNCTION
```

```
TCGA-2Y-A9GS-01A              0.43913538
TCGA-2Y-A9GT-01A              0.08577951
TCGA-2Y-A9GU-01A              0.07839606
TCGA-2Y-A9GV-01A              0.15052318
TCGA-2Y-A9GW-01A              0.58513250
```

4 Visualize TPAC scores

Visualize the TPAC scores in the **S** matrix as a heatmap (this is generated using similar logic as the heatmaps included in the main manuscript.

```
> library(gplots)
> my_palette = colorRampPalette(c("steelblue", "seagreen3",
+                                 "white", "orange", "orangered"))(n = 299)
> breaks = 300
> heatmap.2(t(tpac.out$S),
+           col = my_palette, dendrogram="both", na.rm=T,
+           symm=F, scale = "none", trace = "none",
+           xlab=NA, ylab=NA, labCol=NA, sepcolor="white",
+           sepwidth=c(0, .2), symkey=F,
+           Rowv=T, Colv=T,
+           breaks=breaks, margins=c(2,27),
+           key.title=NA, key.ylab=NA, key.xlab=NA,
+           key.ytickfun=function() {
+             return(list(labels=FALSE, tick=FALSE))
+           },
+           lwid=c(.5,4), lhei=c(.5,4), main = NA)
```

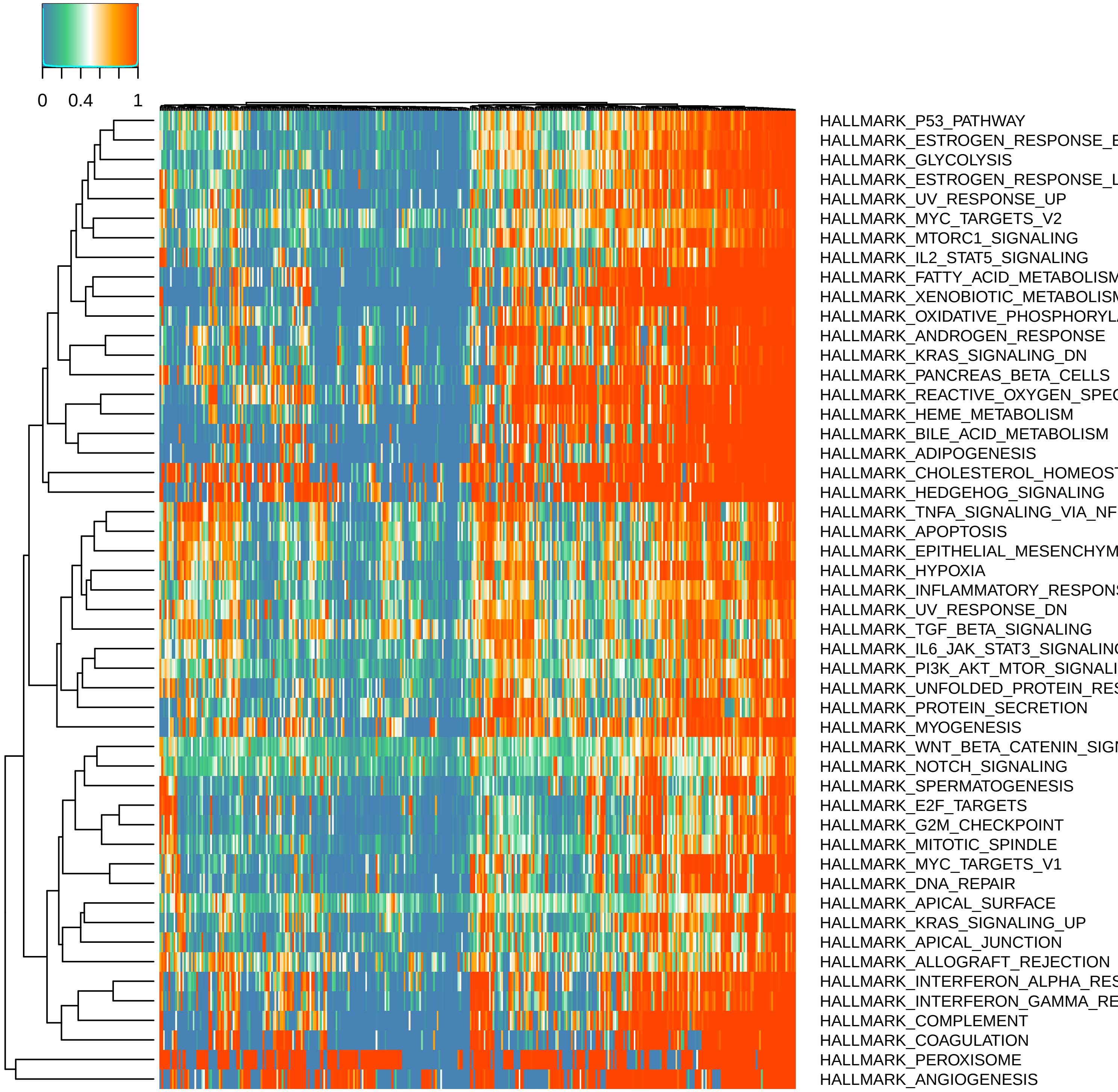

References

[1] Cancer Genome Atlas Research Network, Weinstein, J.N., Collisson, E.A., Mills, G.B., Shaw, K.R.M., Ozenberger, B.A., Ellrott, K., Shmulevich, I., Sander, C., Stuart, J.M.: The cancer genome atlas pan-cancer analysis project. Nat Genet **45**(10), 1113–20 (2013). doi:10.1038/ng.2764

[2] Uhlen, M., Zhang, C., Lee, S., Sjöstedt, E., Fagerberg, L., Bidkhori, G., Benfeitas, R., Arif, M., Liu, Z., Edfors, F., Sanli, K., von Feilitzen, K., Oksvold, P., Lundberg, E., Hober, S., Nilsson, P., Mattsson, J., Schwenk, J.M., Brunnström, H., Glimelius, B., Sjöblom, T., Edqvist, P.-H., Djureinovic, D., Micke, P., Lindskog, C., Mardinoglu, A., Ponten, F.: A pathology atlas of the human cancer transcriptome. Science **357**(6352) (2017). doi:10.1126/science.aan2507
