# Supplementary material for: Tissue-adjusted pathway analysis of cancer (TPAC): A novel approach for quantifying tumor-specific gene set dysregulation relative to normal tissue
Source: PLoS Comput Biol. 2024 Jan 11;20(1):e1011717. doi: 10.1371/journal.pcbi.1011717 (PMC10807770; doi:10.1371/journal.pcbi.1011717)
Supplement: Attachment — Submitted filename: TPAC_response_to_comments.pdf [file pcbi.1011717.s003.pdf]

Response to reviewer comments for "Tissue-adjusted pathway analysis of cancer (TPAC)"

H. Robert Frost

We appreciate the thoughtful, detailed and very helpful feedback from the reviewers. Responses to each of the reviewer's comments are outlined in the sections below (reviewer comments are in bold and our response follows in normal font). In addition to the revised manuscript and SI, the resubmission also includes a file showing the differences between thes revised and original manuscript, as computed via latexdiff.

We believe that addressing the reviewer's comments has substantially improved the revised manuscript and utility of the TPAC method by:

- *Improving manuscript organization/clarity:* In response to comments from all three reviewers, a number of modifications were made to improve the organization and clarity of the manuscript. Relevant changes include the creation of a separate Discussion section, more detail regarding the rationale and approach for the various analyses, regeneration of figures using a color blind friendly palette, migration of some figures to the SI, and expanded literature review.
- *Publishing the TPAC R package on CRAN:* In response to the comment from Reviewer 1, the TPAC R package (and associated TPACData package) are now available via the CRAN repository (https://cran.r-project.org/web/packages/TPAC/index.html).
- *Integration of the GRAPE method for comparative evaluation:* Following the suggestion from Reviewer 2, we have included results from the GRAPE method [1] in all of the comparative evaluations (see new Figures 3-5 and SI Figures S2, S5 and S7).
- *Integration of decoupleR results:* Motivated by the comment from Reviewer 3, we have used the decoupleR method [2] to generate transcription factor (TF) activity estimates for each tumor in the analyzed TCGA cohorts and have included new results that explore the association between TF activity and TPAC scores (see new section "Association between TPAC scores and transcription factor activity illuminates the regulatory impact of tumorigenesis", new Figure 8 and SI Table S2 and Figures S9-S11).

1 Reviewer 1 Comments

Robert Frost presents a new single sample pathway analysis method for the analysis of gene expression data from 21 TCGA cancer types. The method quantifies pathway dysregulation in individual TCGA tumors when compared to the pathway activity in normal tissue. The method is implemented in R and made available via an R package on the author's homepage. The data shows that the method controls the type I

error rate in simulated data, and when compared to existing single-sample enrichment methods, TPAC produces stronger associations with clinical correlates. Strengths include method simplicity and apparent effectiveness in detecting associations between pathway activity and tumor stage and patient prognosis. Weaknesses include that the method seems to be specifically designed for TCGA data and it is not clear how the method would generalize to cancer gene expression data from other sources. Also, the organization of the manuscript needs to be improved.

1. Reviewer 1 Comments on Manuscript Organization

   (a) **It is unusual to refer to display items in the Introduction. Table 1 and the corresponding paragraph referring to Table 1 should be moved to the beginning of the Results section**

   Thank you for raising this issue. We had included Table 1 in the introduction rather than the results section since it represents a synthesis of information from two tables in one of our prior papers, i.e., it is technically part of the background/motivation rather than a new result. Although we could simply reference the original tables rather than reproduce the information in this paper, we felt the content was important for understanding the motivation for the TPAC method (i.e., an enumeration of the target TCGA cancer types and associations between normal tissue specificity and both cancer/normal differential expression and cancer survival) and didn't want to force readers to retrieve and read the earlier paper to review that content.

   Although we have not made any changes to the table or associated text in this revision, if the reviewer feels strongly that this table should not be included in the introduction, we are open to either removing it entirely (and just referencing that prior paper) or moving it to the results section; our preference in this case would be to remove it since it is not a new result and so does not seem appropriate to include within the results section.

   (b) **In agreement with the journal guidelines, Results and Discussion should be divided into two sections. Currently there is no real discussion, the section is reporting merely on results, with no contextualization or interpretation guidance for the reader. The Discussion section is a very important section for most readers, providing an overview, linking the different parts of the manuscript, and helps to embed the findings in the existing literature.**

   The reviewer raises and excellent point and we have separated the Results and Discussion sections with an expanded discussion content to provide a more detailed synthesis/interpretation of the presented results.

   (c) **There are currently too many Figures (10). Consider creating composite figures (ie. thematically grouping figures 3 and 4 as subfigures A and B of one figure; and analogously for eg Figure 5 and 6). Or consider moving 3-5 figures to the Supplement.**

   We thank the reviewer for identifying this issue. Rather than combine the Q-Q plots and heatmaps (which we believe will make the heatmap text more challenging to read), we decided to move the TPAC result heatmaps for cancer prognosis (originally Fig 4, now S4) tumor stage (originally Fig 6, now S6) and lymph node stage (originally Fig 8, now S8) to the SI.

   (d) **Availability + Implementation is currently pointed out at 5 different places in the manuscript (abstract, intro, method, conclusion, data availability).**

Although it is helpful to rather mention availability too often than too less, the typical place to mention availability + implementation would be Methods and in a dedicated availability paragraph at the end of the manuscript.
The reviewers makes an excellent point and we have removed that language from the Abstract, Introduction and Conclusion sections. In response to major comment #7 from Reviewer 2, we have added an explicit "TPAC implementation" section to the Methods that provides an overview of the TPAC R package. More extensive availability and implementation information is also provided in the "Data availability" section at the end of the manuscript.

2. Reviewer 1 Comments by Section

   (a) **Title**

      i. **The title is currently a bit bare bone. Maybe a more accessible title could be: "Tissue-adjusted pathway analysis of cancer (TPAC) increases association strength with tumor stage and patient prognosis" or something along these lines that would allow a reader to immediately grasp the benefits of the method in a cancer expression data analysis setting.**
      Thank you for the suggestion. We have updated the title to the more descriptive "Tissue-adjusted pathway analysis of cancer (TPAC): a novel approach for quantifying tumor-specific gene set dysregulation relative to normal tissue".

   (b) **Introduction**

      i. **The method is currently branded as a pathway analysis method, but it seems to be generally applicable to gene set testing (ie also to gene sets that are not pathways). Also: pathway analysis implies for many readers a certain extent of incorporation of regulatory relationships between the genes in a pathway (= topology- or network-based methods) which is not the case here. This distinction should be made clearer in the first paragraph of the introduction and throughout the manuscript.**
      Thank you for the comment. The reviewer is correct that the TPAC method is generally relevant to all gene sets whether they are associated with unstructured sets (e.g., Gene Ontology terms) or pathways with a known regulatory topology (e.g., Reactome). A similar pathway vs. gene set comment was raised by Reviewer 2 (see minor comment 1). We have clarified this issue in the first paragraph of the revised Introduction. We have also replaced most uses of "pathway" in the manuscript with "gene set" to reduce confusion on this issue.

      ii. **Claim: Ref 19 shows that "the ratio of gene expression in a specific tissue or cancer relative to the mean across multiple tissues or cancers, can be used to improve survival analysis, the comparative analysis of distinct cancer types, and the analysis of cancer/normal tissue pairs" – how precisely does it improve these three analysis types?**
      Thank you for the question. Normal tissue specificity (i.e., the ratio of gene expression in a given normal tissue relative to the average across a large group of tissues) can be leveraged to improve these three types of analyses due to the association that tissue specificity has with cancer survival and cancer/normal differential expression (this is captured in Table 1, which is a reproduction of results from our prior paper, which was ref 19 in the original manuscript and is now ref 21).

Specifically, normal tissue specificity has a positive association with cancer survival (i.e., elevated expression in a tumor of genes that are specific to the associated normal tissue is associated with increased survival); we used this association in our prior paper (see https://doi.org/10.1371/journal.pcbi.1009085) to design a hypothesis weighing/filtering approach that improves power to detect survival associations (this is detailed in the "Use of normal tissue-specificity to improve cancer survival analysis" section in that paper). Genes that are specific to a given normal tissue are also more likely to be down-regulated in the associated cancer; we used this association to design a gene set filtering approach that improved power to detect gene sets differentially expressed in cancer and normal tissues (this is detailed in the "Use of normal tissue-specificity to improve the comparative analysis of normal and neoplastic tissue" section in that paper). For comparative analysis of different cancer types, normal tissue specificity was leveraged to find expression differences between the cancers that are independent of differences that exist between the normal tissues (this is detailed in the "Using normal tissue gene activity to improve the comparative analysis of cancers" section of that paper).

We have expanded the text in the revised Introduction to provide more context on these findings and direct interested readers to our prior paper (now ref 21) for full details.

iii. **“Specifically, we found that genes enriched in normal tissues are more likely to be down-regulated in the associated cancer with elevated expression associated with a favorable cancer prognosis” -> This sentence is difficult to parse. Consider splitting into two sentences: 1) Genes that are highly-expressed in normal tissue are typically down-regulated in cancer; 2) elevated expression of these genes (in cancer?) is typically associated with improved prognosis. “Genes enriched” -> “Highly-expressed genes” or “Genes with increased expression”.**

Thank you for the suggestion. We have split up that sentence per the recommendation.

iv. **Table 1: see comments for the Results section**

Please see responses to the relevant comments for the Results section.

v. **“has an accurate gamma approximation” -> follows approximately a gamma distribution**

The relevant text has been updated.

vi. **“characterize the performance of TPAC” -> evaluate/assess the performance of TPAC**

The relevant text has been updated.

vii. **Consider moving pointers to supplement and R package to the corresponding places in Methods and Results**

As noted above, we have removed language pointing to the SI and R package from the Abstract, Introduction and Conclusion sections.

(c) **Methods**

i. **Why using normal tissue expression data from HPA as opposed to expression in adjacent normal tissues available from TCGA? How are batch effects taken into account when substracting normal tissue expression assayed by HPA from cancer tissue expression assayed by TCGA? These two might reside on somewhat different scales due to batch effects.**

The reviewer asks a great question. The use of HPA normal tissue expression data rather than data from TCGA tumor adjacent normal tissue was primarily done to avoid the impact of field cancerization effects. Regarding batch effects, we followed by approach taken by the HPA team for performing a comparative analysis of their normal tissue RNA-seq data against TCGA RNA-seq data (see their pathology atlas Science paper at https://www.science.org/doi/10.1126/science.aan2507). Specifically, the HPA group reprocessed their normal tissue RNA-seq data using the same computational pipeline used for processing the GDC TCGA RNA-seq data. The HPA group shared this specially processed HPA RNA-seq data with us and we have made that data available via the TPAC paper website (https://hrfrost.host.dartmouth.edu/TPAC/); a sumarized version of this data is also integrated into the TPACData R package and leveraged by the TPAC package. Although the use of a common processing pipeline does not necessarily adjust for all batch effects, this will always be a potential issue when users attempt to apply the TPAC method to non-TCGA cancer expression data. In this context, our use of HPA-based normal tissue data for the analysis of TCGA data provides support for the generalization of the TPAC alysis results shown in this paper to non-TCGA cancer transcriptomic datasets, i.e., if the independent HPA normal tissue data is effective for the analysis of cancer expression data from the TCGA it should also be effective for other cancer datasets assuming a common processing pipeline is used. Generalizability would be less certain if TCGA normal tissue data were used for the analysis of TCGA tumor data. A more detailed discussion of the limitations associated with the use of HPA normal tissue data in this context can be found in the Limitations section of our prior paper (ref 21, https://doi.org/10.1371/journal.pcbi.1009085).

(d) **Results**

i. **Table 1: "TCGA abbrev." -> TCGA disease code**
This had been updated.

ii. **Table 1: It might make sense to restrict the computation of the Spearman rank correlation rho(cancer/norm) to something like the 500-1000 top genes, as computing correlations across something like 20k genes has the potential to dilute signals that are apparent on relevant subsets of the full gene vector.**
We thank the reviewer for the suggestion. Given that this table is a reproduction of results from a prior published work, we would like to keep the current correlation values for consistency with that paper.

iii. **Table 1: Gene weights are computed as the log fold-change of the mean expression in the normal tissue to the mean in all normal tissues. How many samples are there for each normal tissue? Or is there only one sample per tissue? If there were substantially different numbers of samples between normal tissues, taking the mean of all normal tissues could introduce a compositionality effect, where the normal tissues with many more samples would drive the resulting weights. That is similar to differential expression testing between clusters in single-cell RNA-seq data that is used for the identification of marker genes for each cluster.**
The reviewer asks a good question. In this case, the HPA normal tissue RNA-seq data is based on 3 healthly tissue samples per tissue type and all of the HPA normal tissue specificity data/findings are based on the average across these samples. For

the findings in Table 1 and results in this paper, we used the mean expression for each tissue type so effectively treated each normal tissue type as having a single sample. The tissue-specificity values are therefore not impacted by the type of unbalanced sample size issues the reviewer mentioned (though they are impacted by the fact that any tissue type not associated with one of the analyzed cancers is not considered when computing tissue-specificity).

iv. **Figure 1: Type I error control and power analysis is carried out on simulated multivariate normal data, but there is no justification or reference to the literature that this is a good model for real data, in particular FPKMs as available from TCGA that TPAC is applied to in the manuscript**
The reviewer raises and excellent point and we have updated the type I error and power simulation analysis to use simulated count data normalized by library size to mimic the TCGA FPKM data. Specifically, the data counts are simulated as $Poisson(\lambda = 5)$ and then normalized by library size (as opposed to simulating as independent $\mathbb{N}(0, 1)$). To quantify emprical power, the unnormalized counts are inflated prior to normalization; to avoid library size normalization from eliminating the DE signal, we simulated 100 total genes with a gene set of size 20 (i.e., counts are only inflated for the 20 genes in the set). Type I error control is also approximately maintained in this case (0.0531) with a roughly equivalent power curve as generated by the original Gaussian model. The fact that type I error is slightly elevated in this case likely stems from the fact that the squared Mahalanobis distances under the null are only approximately fit by a gamma distribution.

v. **Figure 2: It might make sense to label the gene set clusters in the heatmap as the main text refers to some of them in a not necessarily obvious way ("pathways related to immune signaling", "dysregulation among proliferation pathwats")**
Thank you for the suggestion. We have added annotations for the four main tumor clusters to the bottom of Figure 2 with definitions in the legend that match the text and cluster IDs in the manuscript text, i.e. 1: overall dysregulation across all pathways, 2: minimal dysregulation, 3: immune signaling dysregulation, and 4: proliferation dysregulation.

vi. **Figure 2: It might make sense to spread out the heatmap over the full page width to be able to see whether there are any patterns in the tumor type clustering. From what I can see in the dense display, it seems there is little grouping by tumor / tissue type, which would argue against tissue-specific pathway-dysregulation? This could be discussed.**
Thank you for the suggestion. Unfortunately, attempting to set the figure width to the full paper width causes part of it to be lost when using the the PLOS latex template (it is currently set to full text width). Fortunately, if the paper is accepted, the the official online version will allow readers to open and zoom into the figure as desired so should be able to explore the specific distribution of cancer types across the heatmap clusters. We are happy to explore additional formatting/submission changes to make it easier to resolve the details in this figure during the review process (e.g., include full resolution versions of the figures a separate review-only files).
The reviewer also asks a good question regarding the lack of cancer type clustering in this heatmap. A key thing to note in this context is that the TPAC method

measures dysregulation for each tumor with respect to mean expression in the associated normal tissue and should therefore generate scores that eliminate the expected tissue-based differences. In other words, the scores are expected to capture a tissue-independent pattern of gene expression dysregulation so a lack of clustering by cancer type is the desired output. This issue has been expanded upon in the new discussion section.

vii. **Figure 3, 5, and 7: Whether or not one accounts for tissue-specificity in TPAC seems to hardly make a difference. Why is that? Discussion point.** The reviewer raises a good point. While we agree with the reviewer that the non-tissue specific version of TPAC accounts for most of the improvement in TPAC performance over other techniques, we would argue that the adjustment for tissue-specific does lead to a non-trivial improvement in the number of significant findings for the association with progression free interval (Figure 3; increase in tests with FDF ≤ 0.1 from 247 to 286 or a 16% improvement) and for the association with lymph node stage (old Figure 7, new Figure 5; increase in tests with FDF ≤ 0.1 from 118 to 173 or a 47% improvement). However, it is worth noting that adjusting for tissue specificity actually leads to a decrease in the number of significant associations with tumor stage (old Figure 5, new Figure 4; decrease from 299 to 265 or a 11% decline). We have explored this issue in more detail in the new discussion section. In general, the counts of significant associations are estimates aggregated over all of the analyzed cancer types and all of the Hallmark pathways, so have significant uncertainty. Determining the precise biological mechanism underlying the trends is also very challenging given the heterogeneity between different cancer types and pathways (as reflected in SI Figures S3-S8).

viii. **Figure 3, 5, and 7: TPAC seems to yield more significant results than existing methods in terms of providing lower p-values? Why is that? Is that always beneficial or might this point at times to the method being too liberal in certain scenarios? Discussion point.**
The reviewer makes an excellent point and more significant associations (i.e., an assumed decrease in the type II error rate) is only beneficial if it is not accompanied by an increase in the type I error rate. While it is biologically plausible that changes in survival and cancer stage are associated with increased gene expression dyregulation relative to health tissue, the ground truth is not known in this context. We have included a discussion of this limitation of the reported results in the new discussion section.

ix. **Figure 3, 5, and 7: The number of tests with FDR < 0.1 are shown in brackets. It would be helpful to also have the total number of tests eg in the figure caption.**
Thank you for the suggestion. We have added to the total number of hypothesis tests (1,050) used for computation of the FDR values to the Figure captions (note that these are now Figures 3, 4 and 5).

x. **Figure 4, 6, and 8: Would it make sense to cluster the heatmaps to groups of tumor types and groups of hallmark sets that display similar behavior?** The reviewer asks a good question. For these heatmaps, we had not performed clustering-based row/column reordering to make it easier to compare relative values for specific pathways/cancer types between the separate heatmaps for PFI, tumor stage and lymph node stage. Our inclination is to retain the non-clustered structure

for these heatmaps (which are now all in the SI) but would be happy to make that change if the reviewer felt the clustering structure would be more useful to readers than ease of comparative evaluation between the heatmaps.

xi. **Figure 4, 6, and 8: Typo Figure 4: "rations" -> ratios**
Thank you for noticing this. The caption text has been fixed (note that this figure is now in the SI as Figure S4).

3. Reviewer 1 Comments on TPAC R package

(a) **Availability: TPAC is implemented as an R package that is available for download from the author's homepage. I would strongly encourage making the package available through Github to enable routes for direct installation from github, as well as collaborative elements such as open issue tracking and pull requests. On the other hand, there are existing repositories such as CRAN for R packages in general and Bioconductor for bioinformatic R packages in particular that provide for adherence to R package standards, stable availability, and continuous integration with the R/Bioconductor package ecosystem. I would strongly encourage a submission of this package to Bioconductor.**

Thank you for raising this issue and we also strongly support the use of public repositories like CRAN and Bioconductor. Our original plan had been to release the stable version of this package on CRAN (that is where we typically release all of our R packages) and have gone ahead and published v0.2.0 of the TPAC package (and v0.1.0 of the associated TPACData package) on CRAN for this revision. The paper has been updated to refer to CRAN vs. the paper website for the R package.

(b) **Package DESCRIPTION file: Doesn't need to "depend" on MASS, seems sufficient to "import" from MASS**

Thank you for the recommendation. This has been changed in the 0.2.0 version of the package.

(c) **I could install and use the package as described. However, I encountered the following error in example code of 'tpacForCancer' and 'tpacForCollection': ... Error in stats::optim(x = c(0, 0, 0, 0, 1.01289132327529, 0, 0, 0, 0, : L-BFGS-B needs finite values of 'fn' Error in stats::optim(x = c(0, 0, 0, 0, 0, 0, 0, 0, 0, 0), lower = 0.01, : non-finite value supplied by optim ...**

Thank you for catching this error in the usage example. This was caused by calling fitdistr() to estimate the null gamma distribution on a vector containing 0 elements. For experimental data and real gene sets, zero squared Mahalanobis distances for the positive/negative deviations are very unlikely, however, for the simulated data and 5 member gene set used in the documentation R code, a zero squared distance is quite common with the resulting error. This has been fixed in the 0.2.0 version of the TPAC R package now on CRAN. Specifically, the gamma distribution is only fit on the non-zero null squared Mahalanobis distances (this is also clarified in the methods text). If there are not at least 2 non-zero distances across all samples, then it silently sets all of the associated CDF values to 0 (this occurs for set 2 in the tpacForCancer() usage example for the positive deviation scores).

2 Reviewer 2 Comments

The paper "Tissue-adjusted pathway analysis of cancer (TPAC)" describes a pathway-analysis method. It uses the normal tissue-specificity of human genes to compute a robust multivariate distance score that quantifies pathway dysregulation in profiled tumors. The author addresses an important issue of highlighting pathways to study dysfunctionalities in human tissues, specifically in cancer.

2.1 Reviewer 2 Major Comments

1. **The literature is not up to date. There are a few cited papers that were published after 2020, the latest paper cited was published in 2021. It is recommended to further explore and mention more recent studies in the field. For example, the author claimed that "current approaches leverage just tumor-specific genomic data and do not take into account gene activity in the associated normal tissues". See for example, "Tissue-specific pathway association analysis using genome-wide association study summaries", Bioinformatics 2017.**

   The reviewer raises an excellent point and we have updated the revision to reference the indicated 2017 Bioinformatics paper "Tissue-specific pathway association analysis using genome-wide association study summaries" [3], the 2017 BMC Bioinformatics paper for the GRAPE method [1] mentioned in major comment #2 below and two more recent papers that explore tissue-specific biological processes activity:

   - 2023 Nucleic Acids Research paper "ProAct: quantifying the differential activity of biological processes in tissues, cells, and user-defined contexts" [4]
   - 2022 Bioinformatics paper "The differential activity of biological processes in tissues and cell subsets can illuminate disease-related processes and cell-type identities" [5]

2. **The TPAC method is compared to GSVA, ssGSEA [28] Lee et al., [29], published in 2013, 2009, 2008. Comparison should be made to more recent methods, such as "GRAPE: a pathway template method to characterize tissue-specific functionality from gene expression profiles", BMC Bioinformatics 2017.**

   We thank the reviewer for the suggestion and have updated all of the comparative results to include the GRAPE method. Similar to TPAC, GRAPE is a self-contained method (i.e., only considers the genes within the target set) and, importantly, computes sample-level gene set scores by comparing the set-specific expression profile for a sample to a reference profile. Although the original GRAPE paper did not include an analysis where tumor expression data was scored relative to a normal tissue reference, we were able to perform exactly that type of analysis using the GRAPE R package implementation for a competitive evaluation against TPAC. While TPAC is superior to GRAPE for cancer prognosis prediction (GRAPE performance in this case is similar to the other comparison methods) and GRAPE is unable to perform some of the analyses supported by TPAC (e.g., inference on individual scores, adjustment for tissue-specificity, and generation of separate scores for up and down-regulation of gene expression), the GRAPE scores do have a stronger association with cancer stage (both tumor and lymph node).

   An overview of the GRAPE method, details regarding how the GRAPE method was executed, and key differences between GRAPE and TPAC are now detailed in the "Comparison methods" subsection of "Methods and materials". Figures 3, 4 and 5 in the main manuscript and

SI Figures S3, S5, S7 have been updated to include GRAPE results. A pan-cancer heatmap of GRAPE scores has been added to the SI as a new Figure S2. A discussion of the comparative results is contained in the revised "Results" and "Discussion" sections.

3. **The figures are unclear and should be reorganized in a way that allows readers to compare different cases. It is also recommended to elaborate more on the legend about the plots axes, the colors etc. None of the figures can stand on their own and could not be understood based on the legend. Also, if possible, consider uniting several figures into one with several panels. Fig. 2 is referred to as showing four clusters, but they are unclear.**

   Thank you for the comment. Comments #1.c and #2.d.iv through #2.d.xi by Reviewer 1 raised similar concerns regarding figure content and organization. In particular, in comment #1.c, Reviewer 1 also recommended that some of the figures either be combined into multi-panel figures or moved to the SI (we opted to move some into the SI; see our response above for rationale and details). Reviewer 1 also noted in comment #2.d.v that the four tumor clusters mentioned in Figure 2 are not clearly marked (we have updated Figure 2 to identify those clusters on the bottom of the heatmap). In specific response to this comment, we have expanded the figure caption text so that each figure can be more easily understood without reference to the manuscript text.

4. **The author claims that the TPAC method performed better than the 3 other methods. What were the comparison criteria? Has TPAC preformed significantly better? How was it measured? What were the differences?**

   Thank you for the question. TPAC was originally compared against GSVA, ssGSEA and the z-scoring method of Lee et al.; in response to your comment #1 above, the GRAPE method has been included in this revision. The comparative evaluation focused on the association between the single sample gene set scores generated by these techniques and three different measures of cancer progression/severity: progression free interval (PFI), tumor stage and lymph node stage. Under the assumption that cancer progression will impact the transcriptional activity of important cellular pathways (as represented by the MSigDB Hallmark gene sets), we expected to find statistically signficant associations between single sample scores for many of the Hallmark gene sets and PFI and tumor/lymph node stage. We therefore compared the techniques based on the overall distribution of association p-values (as computed using a Cox proportional hazards model for PFI and Wilcoxon rank sum test for tumor/lymph stage and visualized using Q-Q plots) as well as the number of associations for each technique that were significant at a FDR level of 0.1. As shown in revised Figures 3, 4 and 5, TPAC scores have the most significant associations with PFI (286 vs. a range of 164-191 for the other techniques) and, though the relative gap is not as large, GRAPE scores have the most significant associations with tumor stage (330 vs. a range of 104-299) and lymph node stage (216 vs. a range 108-173).

   When considering the relative benefits of TPAC vs. the other techniques, it is important to keep two items in mind:

   - Some features of TPAC (e.g., single sample inference) could not be assessed via a direct comparative evaluation since they are not supported by the other methods. For single sample inference, we therefore independently evaluated TPAC with relevant results shown in revised Figures 1, 6 and 7.

- The version of the GRAPE method that was used in the comparative evaluation (i.e., the use of HPA normal tissue expression as the reference profile) is distinct from the version detailed in the GRAPE paper. Although the GRAPE paper included TCGA analysis results, the method was not specifically optimized for the analysis of cancer data and the reported analyses did not score tumor expression data relative to the associated normal tissue. Importantly, the strong performance of both TPAC and GRAPE relative to GSVS, ssGSEA and z-scoring for the survival and cancer stage analyses is largely due to the comparison of cancer gene expression to associated normal tissue expression. This use of associated normal tissue data was not explored in the original GRAPE paper and is an important part of the contribution of the TPAC manuscript.

The revised manuscript incorporates the above discussion in the updated Methods, Results and Discussion sections.

5. **t is not clear how the analyses that are described in the results section lead to the conclusions. More efforts should be made to clearly explain the rationale of each of the analyses and the conclusions that were made. It is also recommended that the titles for each section in the results will indicate on the conclusion of the analysis, rather than description (which can be used for figure names, for example).**

   Thank you for the suggestion. We have updated the "TPAC analyses" subsection at the end of the Methods to more clearly state the rationale for each analysis. We have also created a separate "Discussion" section (previously, Results and Discussion were combined), which provides more detail regarding interpretation of the results. We have also implemented your excellent suggestion to rename the titles of the Result subsections to more clearly indicate the conclusion/interpretation; these titles are now:

   - Inference with TPAC scores maintains type I error control
   - TPAC scores reveal pan-cancer landscape of gene set dysregulation
   - TPAC scores have more significant associations with cancer prognosis than scores generated by comparative methods
   - TPAC scores are strongly associated with tumor and lymph node stage
   - Sample-level inference on TPAC scores highlights the significant elements of pan-cancer gene set dysregulation
   - TPAC score statistical significance effectively stratifies patients according to cancer prognosis
   - Association between TPAC scores and transcription factor activity illuminates the regulatory impact of tumorigenesis

6. **The author used rich and diverse analyses. However, the methods should be written in a way that could be reproducible. It might be helpful to summarize the different parameters in a table with their meaning so readers can follow more easily.**

   We thank for the reviewer for their comment. The revised manuscript includes more detail regarding how each of the presented analyses was performed in both the "TPAC analyses" section in the Methods, the various Results subsections and the SI Methods. These details

along with the the release of the TPAC R package on CRAN (which includes a vignette that illustrates the use of the TPAC method to analyze TCGA liver cancer RNA-seq data using MSigDB Hallmark gene sets) should provide interested readers with sufficient guidance to apply TPAC to cancer gene expression data and perform analyses similar to those presented in the paper. Although there is a table in the SI that contains various summary statistics for the analyzed TCGA cohorts, we are uncertain how to construct a table of analysis parameter values would be easy for readers to understand/leverage but are happy to explore that type of table (or another presentation) if reviewer believes that the current methods information is still insufficient to support use of the TPAC technique by other researchers.

7. **It will be helpful to provide documentation and a description of the functions and methods available on the R package.**

   Thank you for the suggestion. We have added a new "TPAC implementation" subsection to the Methods that provides an overview of the TPAC R package and the associated vignette that illustrates the application of TPAC to TCGA liver cancer RNA-seq data using MSigDB Hallmark pathways. Detailed documentation and usage examples for all of the TPAC methods is available via the package documentation on the CRAN website (see https://cran.r-project.org/web/packages/TPAC/index.html).

2.2 Reviewer 2 Minor Comments

1. **The pathways data used in the study are taken from MSigDB, which holds gene sets collections. It is important to clarify it as gene set rather than pathways, as the gene sets do not include information on protein-protein interactions or the direction of the pathway.**

   We thank the reviewer for raising this issue, which was also mentioned by Reviewer 1 in their comment # 2.b.i. Although MSigDB uses the "pathway" label for some sub-collections (e.g, C2.CP Canonical Pathways) and includes gene sets from more traditional pathway databases, e.g., Reactome, all of the information in MSigDB is provided as unordered gene sets. We have added language to the first paragraph of the introduction of the revised manuscript and to the "Data sources" section to clarify the distinction between gene sets and pathways and the fact that TPAC does not leverage information about the relationships between gene set members. We have also replaced most uses of "pathway" in the manuscript with "gene set" to reduce confusion on this issue.

3 Reviewer 3 Comments

The author describes a single sample pathway analysis method for cancer transcriptomics data, named tissue-adjusted pathway analysis of cancer (TPAC). This method aims at leveraging information from normal tissue-specificity of human genes to quantify pathway dysregulation in tumor samples. The TPAC method is based on the evaluation of single samples by implementing Mahalanobis multivariate distance measurements on gene sets. The author claims that TPAC performs well both at population and sample-level. Gene sets definitions are inherited by MSigDB. The calculate TPAC pathway scores by performing analyses on gene expression data for 21 solid human cancers from TCGA and associated normal tissues HPA. TPAC seems to outperforms other single-sample enrichments methods, by providing better results with

both patient prognosis and tumor stage. The author also provide an R package (https://hrfrost.host.dartmouth.edu/TPAC) that I did not test. The method is clearly described, and the benchmarks show strong performances. However, a few issues with the approach and the manuscript writing have been identified and they are listed below:

3.1 Reviewer 3 Major Comments

1. **The main point of this paper seems to be the role of gene activity/expression as a proxy of how tumour cells translate genomic changes into functional advantages/malignancies, and the high cell-type specificity of gene expression, which calls for the development of tissue-specific and sample-specific enrichments. While figure 10 is remarkable in terms of how TPAC scores can be used to stratify tumour samples and eventually predict survival, the paper generally misses a more mechanistic discussion, especially in light of the MSigDB-centric view that is proposed. We understand that this paper scope is to describe a method, but once all this work is done, it would be nice to have a final figure with some reconstruction of the regulatory logic the TPAC score is underlying. Is there a set of transcription factors (TFs), besides MYC that explain higher scores? To answer this question, a very simple and immediate approach could be to apply decoupleR to the transcriptomes of samples with significant TPAC scores. Do they converge towards a subset of meaningful TFs? We are not bound to decoupleR and there are several other ways to answer this question, but following this tutorial would provide a potentially very interesting final figure to the paper in a very small amount of time. https://www.bioconductor.org/packages/release/bioc/vignettes/decoupleR/inst/doc/tf_bk.ht**

   Thank you for the excellent suggestion. We have included results from a decoupleR-based TF activity analysis in the Results subsection "Association between TPAC scores and transcription factor activity illuminates the regulatory impact of tumorigenesis" of the revised manuscript with details on the analysis in the Methods section and additional results in the SI. Specifically, we followed the approach in the referenced vignette to compute activity scores for 618 TFs for each tumor in all analyzed TCGA cohorts. We then computed the rank correlation between the activity scores for each TF and TPAC scores for each of the MSigDB Hallmark gene sets. This analysis generated a TF-by-gene set matrix of rank correlations for each of the cancer types (these are visualized for the top 25 TFs for the KIRP and KICH cohorts in SI Figures S10 and S11). To visualize an overall summary, we averaged these correlations across all of the gene sets (this is what is shown in the new Figure 8 in the revised manuscript) or across all of the cancer types (this is shown in SI Figure S9). Although the pattern of association varied significantly between different cancer types and few TFs had a consistent association across all gene sets, the results of the analysis were consistent with known TF/cancer associations and may provide some additional insight into the regulatory mechanisms linked to biological process dsyregulation in cancer.

2. **One of the authors previous work is reported here as reference 11. In figure 1 of reference 11, there is a schematic in which "single-sample pathway enrichment" is reported. Also, Fig.2 of Ref 11 seems to provide similar information with respect to Fig. 6 and 8 of the current paper. We require the author to stress the differences between their previous work and the current manuscript, the advantages of the new method, ultimately comparing old and new results.**

   We appreciate the comment by the reviewer and are happy to hear that our 2018 paper is

still being read! The original reference 11 (now ref 13) is a paper we published in 2018 titled "A multi-omics approach for identifying important pathways and genes in human cancer". While both that paper and TPAC paper performed single sample gene set testing of TCGA transcriptomic data using MSigDB gene sets, the motivation and novel elements of the two papers are very distinct. In particular:

- In Ref 11, we leveraged the existing GSVA method to perform single sample gene set testing of the TCGA gene expression data. In the current paper, we developed a new technique, TPAC, and compared it in performance to existing methods like GSVA.
- In Ref 11, the goal was to evaluate how well somatic alteration data can predict transcriptomic activity as represented by the single sample gene set scores. In this paper, our goal is the development and evaluation of a novel gene set testing method that leverages information about normal tissue specificity and we evaluated the effectiveness of TPAC relative to other techniques like GSVA by computing the association between gene set scores and cancer prognosis, tumor stage and lymph node stage. Although the analyses performed in the 2018 paper regressing gene set scores on somatic alteration data could be repeated using TPAC scores rather than GSVA scores, that type of analysis was not within the scope of the current paper.
- Although both Fig 2 in Ref 11 and Figures 6 and 8 in this manuscript (these are now SI Figures S6 and S8) are gene set-by-cancer type heatmaps, they capture very different statistics. Fig 2 in Ref 11 visualizes predicted R^2 values from a penalized regression of single sample gene set scores on somatic alteration data; Figs 6 and 8 in the original TPAC paper directly visualize the single sample gene set scores.

3.2 Reviewer 3 Minor Comments

1. **Line 13. We understand MSigDB has been used as a reference but there is a vast amount of gene set collections besides MSigDB. We see a value in sticking to a single collection, however, testing the efficacy of the method with other knowledge base would be demanding but not out of scope. Hence, we ask the author to at least mention alternatives to MSigDB that could be exploited when implementing TPAC.**

   The reviewer raises an excellent point. Although MSigDB aggregates a large number of different gene set collections (e.g., Gene Ontology, Reactome, ImmuneSigDB, BioCarta, KEGG, Hallmark, etc.), there are some collections not covered by MSigDB and, most importantly, some of the collections in MSigDB are included in a significantly filtered (e.g., Gene Ontology and KEGG) or simplified version (e.g., loss of pathway topology for Reactome). We have noted these limitations of MSigDB and alternative collection sources in the "Data sources" section of the revised manuscript.

2. **Colours in Heatmaps don't seem colour-blind friendly. This needs to be fixed.**

   Thank you for identifying this issue. We have regenerated the heatmaps using the color blind friendly "viridis" palette.

3. **The following sentence from the abstract "Because the null distribution of the TPAC scores has an accurate gamma approximation, both population and sample-level inference is supported." is repeated equally at line 46 and 310. I understand**

it's a bit of a mantra for the author but I'm quite sure it can be sacrificed or at least modified in the abstract or in the text.

The reviewer makes a good point. We left the original language in the abstract but have removed the version at the end of the introduction and modified the wording in the conclusion.

References

[1] Michael I Klein, David F Stern, and Hongyu Zhao. Grape: a pathway template method to characterize tissue-specific functionality from gene expression profiles. *BMC Bioinformatics*, 18(1):317, Jun 2017.

[2] Pau Badia-I-Mompel, Jesús Vélez Santiago, Jana Braunger, Celina Geiss, Daniel Dimitrov, Sophia Müller-Dott, Petr Taus, Aurelien Dugourd, Christian H Holland, Ricardo O Ramirez Flores, and Julio Saez-Rodriguez. decoupler: ensemble of computational methods to infer biological activities from omics data. *Bioinform Adv*, 2(1):vbac016, 2022.

[3] Wenyu Wang, Jingcan Hao, Shuyu Zheng, Qianrui Fan, Awen He, Yan Wen, Xiong Guo, Cuiyan Wu, Sen Wang, Tielin Yang, Hui Shen, Xiangding Chen, Qing Tian, Lijun Tan, Hong-Wen Deng, and Feng Zhang. Tissue-specific pathway association analysis using genome-wide association study summaries. *Bioinformatics*, 33(2):243–247, Jan 2017.

[4] Moran Sharon, Gil Gruber, Chanan M Argov, Miri Volozhinsky, and Esti Yeger-Lotem. Proact: quantifying the differential activity of biological processes in tissues, cells, and user-defined contexts. *Nucleic Acids Res*, 51(W1):W478–W483, Jul 2023.

[5] Moran Sharon, Ekaterina Vinogradov, Chanan M Argov, Or Lazarescu, Yazeed Zoabi, Idan Hekselman, and Esti Yeger-Lotem. The differential activity of biological processes in tissues and cell subsets can illuminate disease-related processes and cell-type identities. *Bioinformatics*, 38(6):1584–1592, Mar 2022.
